# On-road emissions of passenger cars beyond the boundary conditions of the real-driving emissions test^☆^

**DOI:** 10.1016/j.envres.2019.108572

**Published:** 2019-09

**Authors:** Ricardo Suarez-Bertoa, Victor Valverde, Michael Clairotte, Jelica Pavlovic, Barouch Giechaskiel, Vicente Franco, Zlatko Kregar, Covadonga Astorga

**Affiliations:** a European Commission Joint Research Centre (JRC), Ispra, Italy; b European Commission Directorate-General for Environment, Brussels, Belgium

**Keywords:** Air pollution, Vehicle emissions, Euro 6, RDE, NOx, PN, CO

## Abstract

Passenger cars are an important source of air pollution, especially in urban areas. Recently, real-driving emissions (RDE) test procedures have been introduced in the EU aiming to evaluate nitrogen oxides (NOx) and particulate number (PN) emissions from passenger cars during on-road operation. Although RDE accounts for a large variety of real-world driving, it excludes certain driving situations by setting boundary conditions (e.g., in relation to altitude, temperature or dynamic driving).

The present work investigates the on-road emissions of NOx, NO_2_, CO, particle number (PN) and CO_2_ from a fleet of 19 Euro 6b, 6c and 6d-TEMP vehicles, including diesel, gasoline (GDI and PFI) and compressed natural gas (CNG) vehicles. The vehicles were tested under different on-road driving conditions outside boundaries. These included ‘baseline’ tests, but also testing conditions beyond the RDE boundary conditions to investigate the performance of the emissions control devices in demanding situations.

Consistently low average emission rates of PN and CO were measured from all diesel vehicles tested under most conditions. Moreover, the tested Euro 6d-TEMP and Euro 6c diesel vehicles met the NOx emission limits applicable to Euro 6d-TEMP diesel vehicles during RDE tests (168 mg/km). The Euro 6b GDI vehicle equipped with a gasoline particulate filter (GPF) presented PN emissions < 6 × 10^11^ #/km. These results, in contrast with previous on-road measurements from earlier Euro 6 vehicles, indicate more efficient emission control technologies are currently being used in diesel and gasoline vehicles.

At the same time, the results suggest that particular attention should be given to CO and PN emissions of certain types of vehicles when driven under dynamic conditions, and possibly additional work is necessary. In particular, the emissions of CO (measured in this study during the regulated RDE test, but without an emission limit associated to it) or PN from PFI vehicles (presently not covered by the Euro 6 standard) showed elevated results in some occasions. Emissions of CO were up to 7.5 times higher when the more dynamic tests were conducted and the highest PN emissions were measured from a PFI gasoline vehicle during dynamic driving. Although based on a limited sample of cars, our work points to the relevance of a technology- and fuel-neutral approach to vehicle emission standards, whereby all vehicles must comply with the same emission limits for all pollutants.

## Highlights

• 19 Euro 6b + vehicles, including diesel, gasoline and CNG, were tested on-road.
• NOx, NO_2_, CO, PN and CO_2_ emissions were measured under different driving conditions.
• Euro 6d-TEMP diesels presented lower NOx emissions than pre-RDE Euro 6 diesels.
• CO and PN emissions from gasoline vehicles can be high under certain conditions.

## 1 Introduction

Poor air quality is an important environmental health hazard, resulting in health problems for the population and high costs for health care systems (European Environmental Agency, 2018). More than 95% of the European population living in urban areas is exposed to ambient air concentration levels of particle matter (PM), NOx or O_3_ that are deemed unsafe by the World Health Organization (WHO) (European Environmental Agency, 2018).

Passenger cars are an important source of NOx, CO, volatile organic compounds (VOCs), primary aerosols and secondary aerosol precursors (European Environmental Agency, 2018; Gordon et al., 2014; Platt et al., 2017; Suarez-Bertoa et al., 2015a; Link et al., 2017; Anenberg et al., 2017). Once in the atmosphere, these pollutants play key roles in the formation of tropospheric ozone (O_3_) and secondary aerosols that impair air quality.

Vehicle emission standards were progressively introduced aiming at improving air quality by reducing air pollution at the source. Although the standards have been largely successful, the actual emission reductions were in some instances lower than what would be expected from the gradual decrease in nominal emission limits. Notably, recent studies have shown that the on-road NO_X_ emissions from diesel passenger cars routinely exceeded the emission levels measured during the chassis dynamometer laboratory tests performed according to the New European Driving Cycle (NEDC, superseded by WLTP) (Weiss et al., 2012; Degraeuwe and Weiss, 2017; Baldino et al., 2017; Pavlovic et al., 2018).

The discrepancy between real world emissions and laboratory tests has been documented in various studies (Weiss et al., 2012; Fontaras et al., 2017; Pavlovic et al., 2018). The national investigation programs (BMVI 2016; GOV.UK 2016; RDW and Netherlands Vehicle Authority, 2017; MIT 2017) launched in various European Member States after the diesel scandal have attributed the discrepancy to various causes, including the exploitation of ‘flexibilities’ in the emissions type-approval procedure and some illegal practices. In order to narrow the gap between laboratory emissions and real world emissions, the World harmonized light-duty test procedure (WLTP) and the RDE test procedures—the latter using Portable Emissions Measurement Systems (PEMS)have been recently introduced in the EU (EU 1151/2017). These tests were devised to be more representative of real-world driving conditions than NEDC. Being an on-road test subject to variability in several factors (ambient conditions, driver's behaviour, payload, etc.), RDE accounts for a large variety of real-world driving situations covering a ‘normal driving’ testing space. Regulated RDE tests do however exclude some less frequent driving situations by setting boundary conditions, which define driving situations invalidating an RDE test (e.g., dynamic driving, excessive positive elevation gain, altitude and temperature ranges).

The European RDE regulation defines not-to-exceed (NTE) limits for NOx and solid particle number (PN) emissions, meaning that all valid RDE tests must result in emissions that satisfy those limits. These NTE limits are defined via so-called conformity factors (CFs), which are multipliers of the reference emission limit applicable to the laboratory procedure. The conformity factors for Euro 6d are greater than one to reflect the increased measurement uncertainty of PEMS, and they are subject to periodical review (Clairotte et al., 2018; Giechaskiel et al., 2018c).

Several authors have recently reported emissions, mainly NOx and CO_2_ emissions, from RDE-like test (Kwon et al., 2017; Gallus et al., 2017; Costagliola et al., 2018; O'Driscoll et al., 2018; Ramos et al., 2018; Suarez-Bertoa et al., 2019). Only a few similar studies reported emissions from gasoline vehicles (Pathak et al., 2016; Khan and Frey, 2018; Demuynck et al., 2017), sometimes focusing on PN emissions (Giechaskiel et al., 2015; Ko et al, 2018, 2019 Demuynck et al., 2017). Some studies explored real-world emissions outside RDE boundary conditions. Gallus et al. have recently shown that driving style and road grade have an impact on the CO_2_ and NOx on-road emissions of two diesel vehicles. Costagliola et al., (2018) investigated the impact of road grade on the real driving emissions of CO, NOx, and CO_2_ from two Euro 5 diesel vehicles; Chong et al., (2018) showed how exhaust gas recirculation (EGR) rate and diesel particulate filter (DPF) regeneration events affect gaseous emissions from diesel vehicles.

The present work investigates the emissions of NOx, NO_2_, CO, PN and CO_2_ from a fleet of 19 Euro 6b + vehicles, including diesel, gasoline (GDI and PFI) and CNG vehicles, under different driving conditions. Emissions of the vehicles tested during RDE compliant tests, which act as base line, were compared to emissions obtained during tests that do not fulfill the boundary conditions in terms of dynamicity (excessively dynamic driving), share of operation (too long urban and/or motorway shares), altitude gain (excessive altitude gain), among others. The work does not only shed light on the current state of vehicle emissions under different real world conditions but it is an important source for emission factor development. The obtained emission factor will allow updating current vehicle emissions inventories and provides real world emissions of pollutants that are not included in on-road regulation at the moment (CO and PN from PFI). Moreover, it presents the first results of vehicles type-approved under the most stringent emission standards at the moment (Euro 6d-TEMP) investigated under different real-world driving situations.

## 2 Experimental

The Real Driving Emissions regulation introduced on-road testing with PEMS to complement the laboratory Type I test for the type approval of light-duty vehicles in the European Union focusing on NOx and PN. The RDE, which was introduced in the European Union legislation through several successive regulatory packages (RDE1 (EU Regulation, 2016/427), RDE2 (EU Regulation, 2016/646), RDE3 (EU Regulation, 2017/1154) and RDE4 (EU Regulation, 2018/1832)) is applicable to all new types since September 1^st^ 2017 and to all new vehicles since September 1^st^ 2018. During the phasing-in of the RDE regulation (2017–2019) a temporary conformity factor of 2.1 for NOx tailpipe emissions may apply upon the request of the manufacturer. Vehicles type approved under this requirement fall under Euro 6d-TEMP standard. Based on an amendment done in RDE4, a conformity factor of 1.43 will be applicable for all new types and all new vehicles from January 1^st^ 2020 and January 1^st^ 2021, respectively. This conformity factor requires full compliance with the Euro 6 emission limits (i.e., a conformity factor of 1), but allows a margin of 0.43 to account for the additional measurement uncertainty of PEMS relative to standard laboratory equipment. The margin for particle number was set to 0.5 in RDE3 and it is applicable since September 1^st^ 2018 for all new vehicles. On top of the requirements set at the type approval stage (i.e., before entering the market), RDE4 introduced the In-Service conformity (ISC) procedure for which vehicles need to show compliance with their emission limits throughout their normal life (5 years or 150 000 km, whichever is sooner) under normal conditions of use. ISC will be applicable for all new vehicles since September 1^st^ 2019.

In the present study, we investigated emissions from Euro 6b vehicles and Euro 6c (GV5 and DV7) (hereinafter pre-RDE vehicles). These vehicles were type-approved under the NEDC and did not undergo through on-road testing during type-approval. Their emission limits, based on the NEDC procedure, for vehicle classes M1, N1 Class 1 (same limit as M1) and N1 Class 3 and are summarized in Table 2.

We also investigated emissions from Euro 6d-TEMP type-approved vehicles (DV8-DV10), which underwent through the WLTP and RDE testing during homologation. During the type-approval WLTP the vehicles had to comply with the same emission limits as Euro 6b and with the final PN limit for diesel and GDI vehicles, 6.0 × 10^11^ #/km (see Table 2). During the RDE tests of the type-approval, these vehicles had to comply with the limits and a CF of 2.1 for NOx and 1.5 for PN.

Emissions of NOx, NO_2_, CO, PN and CO_2_ from 19 Euro 6 vehicles (see Table 1 for main characteristics and for further details including brand and model) were comprehensively studied under different driving conditions. The fleet comprises, 8 gasoline (7 Euro 6b and 1 Euro 6c - GV5) 10 diesel (6 Euro 6b, 1 Euro 6c and 3 Euro 6d-TEMP) and 1 Euro 6b CNG light commercial vehicle (hereinafter CNG-LCV). The vehicle DV2-LCV, a diesel Euro 6b N1 Class 1 vehicle, must meet the same emission standards as the other Euro 6b diesel vehicles tested, and therefore it was included with them in the analysis. The vehicles were tested of RDE-compliant tests, which act as baseline, and also during tests that do not fulfill RDE boundary conditions in terms of dynamicity (excessive dynamic driving), share of operation (urban and/or motorway shares above RDE requirements), altitude gain (excessive altitude gain), among others. The vehicles were tested with PEMS over four different pre-defined routes in the Italian region of Lombardy: two fully RDE-compliant (route identifiers RDE-1 and RDE-2) and 2 non-RDE compliant (City-Motorway and Hill) (see Table 3). Average ambient temperature during the entire campaign was 21 °C, where the maximum ambient temperature was 33 °C (GV7) and the minimum 5 °C (GV2). The tests were conducted by technical staff of the Sustainable Transport Unit of the European Commission Joint Research Centre (EC-JRC) between March 2017 and September 2018).

**Table 1:** Vehicle specifications.

Code	Brand	Model	Fuel	Injection	Emission Control system	Reg. Year	Euro standard	Vehicle class	Engine Capacity (cm^3^)	Power (kW)
GV1	Fiat	Panda	Gasoline	PFI	TWC	2016	Euro 6b	M1	1242	51
GV2	Renault	Twingo	Gasoline	PFI	TWC	2017	Euro 6b	M1	999	51
GV3	Audi	A1	Gasoline	DI	TWC	2016	Euro 6b	M1	999	70
GV4	Opel	Astra	Gasoline	DI	TWC	2017	Euro 6b	M1	999	77
GV5	VW	Golf BlueMotion	Gasoline	DI	TWC	2017	Euro 6c	M1	1498	96
GV6	Lancia	Ypsilon	Gasoline	PFI	TWC	2016	Euro 6b	M1	875	63
GV7	Renault	Clio	Gasoline	DI	TWC	2016	Euro 6b	M1	1197	87
GV8	VW	Tiguan	Gasoline	DI	TWC + GPF	2018	Euro 6b	M1	1395	110
DV1	Fiat	500X	Diesel	DI	DOC + EGR + DPF + LNT	2016	Euro 6b	M1	1956	103
DV2-LCV	Peugeot	Partner	Diesel	DI	DOC + EGR + DPF + SCR	2017	Euro 6b	N1, Class 1	1560	73
DV3	Kia	Sportage	Diesel	DI	DOC + EGR + LNT + DPF	2017	Euro 6b	M1	1685	85
DV4	VW	Golf BlueMotion	Diesel	DI	DOC + EGR high/low + LNT + DPF	2015	Euro 6b	M1	1968	110
DV5	BMW	530d - 5 series G30	Diesel	DI	DOC + EGR + SCR + LNT + DPF	2017	Euro 6b	M1	2993	195
DV6	Mercedes-Benz	C220d	Diesel	DI	DOC + EGR + DPF + SCR	2017	Euro 6b	M1	2143	125
DV7	Škoda	Superb	Diesel	DI	DOC + EGR + DPF + SCR	2017	Euro 6c	M1	1968	110
DV8	Peugeot	308	Diesel	DI	DOC + EGR + DPF + SCR	2018	Euro 6d-TEMP	M1	1499	96
DV9	Volvo	XC40	Diesel	DI	DOC + EGR + DPF + LNT + SCR	2018	Euro 6d-TEMP	M1	1969	140
DV10	Ford	Focus	Diesel	DI	DOC + EGR + LNT + DPF + LNT + pSCR	2018	Euro 6d-TEMP	M1	1499	88
CNG-LCV	Fiat	Ducato	CNG	PFI	TWC	2018	Euro 6b	N1, Class 3	2999	100

**Table 2:** Emission limits for positive ignition Euro 6 M1; N1-Class 1 and N1-Class 3 vehicles and compression ignition Euro 6 M1; N1-Class 1 vehicles.

positive ignition Euro 6b, 6c, 6d-TEMP, 6d						
Class	NMHC (g/km)	HC (g/km)	CO (g/km)	NOx (g/km)	PM (mg/km)	PN (#/km)
M1; N1 Class 1	0.068	0.100	1.000	0.060	4.5	6.0 × 10^11^^a^
N1, Class 3	0.108	0.160	2.270	0.082	4.5	6.0 × 10^11^^a^
compression ignition Euro 6b, 6c, 6d-TEMP, 6d						
	–	HC + NOx (g/km)	CO (g/km)	NOx (g/km)	PM (mg/km)	PN (#/km)
M1; N1 Class 1	–	0.170	0.500	0.080	4.5	6.0 × 10^11^

a Applicable only to vehicles using direct injection engines; 6.0 × 10^12^ #/km within first three years from Euro 6 effective dates.^a^

**Table 3:** Trips characteristics. Bold indicates that the value is outside RDE boundary conditions.

	RDE compliant routes		Non-RDE compliant routes			
	RDE-A	RDE-B	RDE-A-Dynamic	RDE-B-Dynamic	City-Motorway	Hill
Trip distance (km)	79	94	79	94	139	61
Average trip duration (min)	98	112	94	104	136	106
Average stop time (%)	20	19	23	19	17	7
Average Urban distance (km)	32	37	31	34	44	61
Average Rural distance (km)	25	27	25	28	18	**-**
Average Motorway distance (km)	22	30	23	32	80	**-**
Urban average Speed (km/h)	29	29	29	31	31	34
Average Urban 95th v*a (m^2^/s^3^)	13	13	20	20	10	9
Average Rural 95th v*a (m^2^/s^3^)	19	17	29	30	19	–
Average Motorway 95th v*a (m^2^/s^3^)	19	21	29	30	18	–
Cumulative positive gain (m/100 km)	760	820	760	820	440	1830
Max trip altitude (m.a.s.l.)	300	415	300	415	295	1088

The vehicles were selected to be a representative sample of the European market for new vehicles. The studied vehicles are a sub-sample of the test fleet selected in the scope of a preparatory study carried out by the JRC to prepare the future market surveillance activity in Europe (as required by the new type approval framework Regulation, 2018/858). In this preparatory study, the vehicles were selected after a risk assessment process including i/the sales numbers per manufacturers collected from different sources (European Environmental Agency and European Automobile Manufacturer Association 2017 databases (European Environmental Agency, 2017; ACEM, 2017)), ii/the vehicle segment size (Thiel et al., 2014), and iii/vehicles' engine and after-treatment main technologies. In order to investigate the two technologies widely implemented by the light-commercial vehicles (i.e., diesel and CNG), two Euro 6b LDVs (DV2-LCV and CNG-LCV) were tested, one equipped with a diesel engine (Class 1 - reference mass < 1305 kg) and one equipped with a CNG engine (Class 3 - reference mass > 3500 kg). More details on the risk assessment process can be found in the JRC 2017 annual report (Clairotte et al., 2018).

The tested vehicles were selected to be representative of the engine and exhaust after-treatment technologies used in the EU for new cars sold between 2016 and 2018. Hence, the tested gasoline vehicles used either port fuel injection (PFI) or direct injection (GDI) technology. One gasoline vehicle (GV8) was equipped with a gasoline particulate filter (GPF). All diesel vehicles were equipped with an exhaust gas recirculation (EGR) system and either a lean NOx trap (LNT), selective catalytic reduction (SCR) or both (DV9) to control NOx emissions. One diesel vehicle (DV10, type-approved to Euro 6d-TEMP) was equipped with a dual LNT and a passive SCR (not requiring urea solution refills).

All vehicles were tested using the applicable laboratory procedures for exhaust emissions, i.e., WLTP (EU 2017/1151) for vehicles DV8, DV9 and DV10, and Type 1 test according to UNECE Regulation 83 for all others (see (Clairotte et al., 2018; Suarez-Bertoa and Astorga, 2018) for a complete description of the tests). The corresponding Euro 6 limits were met in all cases. Compliance with the emission limits over the laboratory test was taken as indication that the vehicles were free of malfunctions that could result in abnormally high emissions. Additionally, an on-board diagnostics (OBD) scan was performed before and after a vehicle was tested.

The measurement of the instantaneous, on-road emissions of NOx, NO_2_, CO, PN and CO_2_ were performed using PEMS. Vehicle DV1 was tested using a Semtech Ecostar system (Sensors, Saline, Michigan, USA – model 2013), and all other vehicles were tested using an AVL MOVE system (AVL, Graz, Austria – model 2016). Both PEMS systems consist of a tailpipe attachment, heated exhaust lines, an exhaust flow meter (EFM), exhaust gas analyzers, a solid particle counter, data logger connected to vehicle network, a GPS and a weather station for ambient temperature and humidity measurements. Both systems measure exhaust gas concentrations of CO and CO_2_ by a non-dispersive infrared sensor, and NO and NO_2_ by a non-dispersive ultra-violet sensor. NOx is calculated by the sum of the concentrations of NO and NO_2_. PN was measured by means of diffusion charge methodology using the MOVE (GV5-GV8, DV3, DV7-DV10) or by condensation particle counter (CPC) using a TSI NPET 3795, modified by HORIBA to reach higher concentrations (GV1-GV4, DV1, DV2, DV4 – DV6 and CNG-LCV). EFM uses a Pitot tube to calculate flow rate. All relevant emissions data were recorded at a frequency of 1 Hz. The PEMS used in the described experimental campaign are routinely validated on the chassis dynamometer as recommended by RDE regulation. Pointner et al., (2017) studied the pressure change up to 3000 m.a.s.l. (700 mbar ab) for an AVL 492 Gas PEMS iS. They found that the instrument performed well under these elevated ambient conditions, presenting a deviation of all measured concentrations within 2% rel. of the measured value.

The current RDE procedure described in the legislation (EU 2017/1154) prescribes a series of requirements and boundary conditions for a test to be RDE compliant. Moreover, in order to be RDE compliant the route use must fulfil a series of requirements. Table 4 summarizes a not exhaustive list of these requirements and boundary conditions for a test to be RDE compliant. N1 vehicles, present some specific requirements that, together with the emission limits for Euro 6 positive ignition N1 vehicles, have been added in the supplementary material. The emission limits for positive ignition N1 Class 3 vehicles (such as CNG-LCV), which are higher than those of M1 and N1 Class 1 vehicles, are presented in Table 2. Some other general aspects were also systematically applied during the testing campaign: vehicles were soaked inside a facility at least 4 h between two consecutive tests; when present, the stop/star option was enabled; the vehicle's battery was not charged between tests; the air conditioning system was consistently set to 21 °C, when possible, or to similar conditions when the vehicle did not offer the automatic temperature setting; the test performed on the previous day was used as preconditioning for the actual test.

**Table 4:** Some of the requirements and boundary conditions for a test to be RDE compliant.

Altitude (m.a.s.l.)	Moderate conditions	0–700
	Extended conditions	700–1300
Ambient temperature	Moderate conditions	0–30 °C
	Extended conditions	−7 – 0 °C and 30–35 °C
Cumulative positive elevation gain		1200 m every 100 km
Altitude difference between start and finish		<100 m
Dynamics	Upper limits	95th percentile of the multiplication of the instant speed and positive acceleration signals as defined in Appendix 7a, Section 4 of RDE 2.
	Lower limits	Relates to the relative positive acceleration as defined in Appendix 7a, Section 4 of RDE 2.
Maximum speed		145 km/h (up to 160 km/h for <3% of motorway driving time).
Payload		Maximum 90% of the maximum vehicle weight (including the mass of the driver and measurement equipment).
Stop percentage		Between 6% and 30% of the urban driving time.
Speed	Average urban speed	15–40 km/h
		above 100 km/h for at least 5 min.
Distance		Urban >16 km; Rural >16 km; Motorway >16 km
Trip Composition		Urban 29–44% of the total distance; Rural 23–43% of the total distance; Motorway 23–43% of the total distance.
Total Trip Duration		90–120 min
Use of auxiliary systems		Operated as in real life use (air conditioning, etc.).

Table 3 summarizes the main characteristics of the four routes used. Route RDE-1 and Route RDE-2 were designed to fulfil all the requirements of the RDE procedure. Route City-Motorway presents a different sequence of vehicle operation (City-Motorway-City-Motorway-City instead of the usual urban-rural-motorway), and urban and motorway shares are longer than allowed by RDE. Moreover, the City-Motorway route was developed to investigate the emissions of the vehicles during prolonged motorway driving, which is a realistic situation for many vehicles of the EU fleet, and also during urban driving after the vehicles and emission control systems have been heat up and conditioned. This second urban section begins ~4000s after the test started, and lasts ~1400s. Route Hill has a positive altitude gain outside RDE boundaries (~1800m), and it comprises only urban operation. The Hill route aimed at examine if the vehicles presented drastic differences in their performance when driving uphill and/or during a long urban section. If this was the case a different engine strategy could have been triggered and a more detailed investigation would had been needed to evaluate if this was allowed in the regulation.

Vehicles were tested fulfilling RDE requirements along route RDE-1 and route RDE-2. Then, they were tested through the two same routes using a more dynamic driving style (i.e., seeking an increase in the 95^th^ percentile of v*a). Even if dynamic tests presented higher v*a, some of these tests fulfilled the Max. 95^th^ percentile of v*a RDE boundary. The higher dynamicity was achieved for example by faster starts after fully stopping the vehicle at traffic lights or engaging lower gears when using manual transmission. All the dynamic tests were performed respecting the Italian traffic code.

The emission factors reported in this paper were calculated by integrating the total mass emissions measured during the test and dividing the obtained value by the driven distance, as estimated from the GPS velocity signal. These are the so-called ‘raw’ emissions (without using the weighting function based on CO_2_ emissions as introduced in the fourth package of the RDE regulation –RDE4) (EU 2018/1832). All the emissions, including cold-start and idle emissions along the test have been included in the data analysis as required by the 4^th^, and last, package of the RDE regulation (EU Regulation 2018/1832).

Furthermore, when vehicles were tested at ambient temperature above 30 °C, or vehicles were tested above 700 m above sea level (Hill route), the measured emissions were not divided by the ‘extended conditions’ factor of 1.6, as would be required by the RDE regulation.

It should be noticed that, as indicated in the Introduction section, Euro standards (even Euro 6d which is the most stringent at the moment) only present on-road real driving emission limits for NOx emissions, from all vehicles, and for PN emissions, from diesel vehicles and GDI vehicles. This means that none of the tested vehicles (except DV8 – DV10) were designed to emit lower NOx and PN than the NTE over an RDE test since they were type-approved before the RDE regulation entered into force. Vehicles, however, have to comply with the limits for criteria pollutants, which does not include PN emissions from non-direct injection gasoline vehicles (i.e., port fuel injection – PFI), under their corresponding type-approval test procedure. PFI vehicles typically produce PN emissions below the PN limit in standard testing or driving conditions. For that reason, only diesel and GDI vehicles were required to meet a PN limit in Europe.

## 3 Results and discussion

**Fig. 1:**
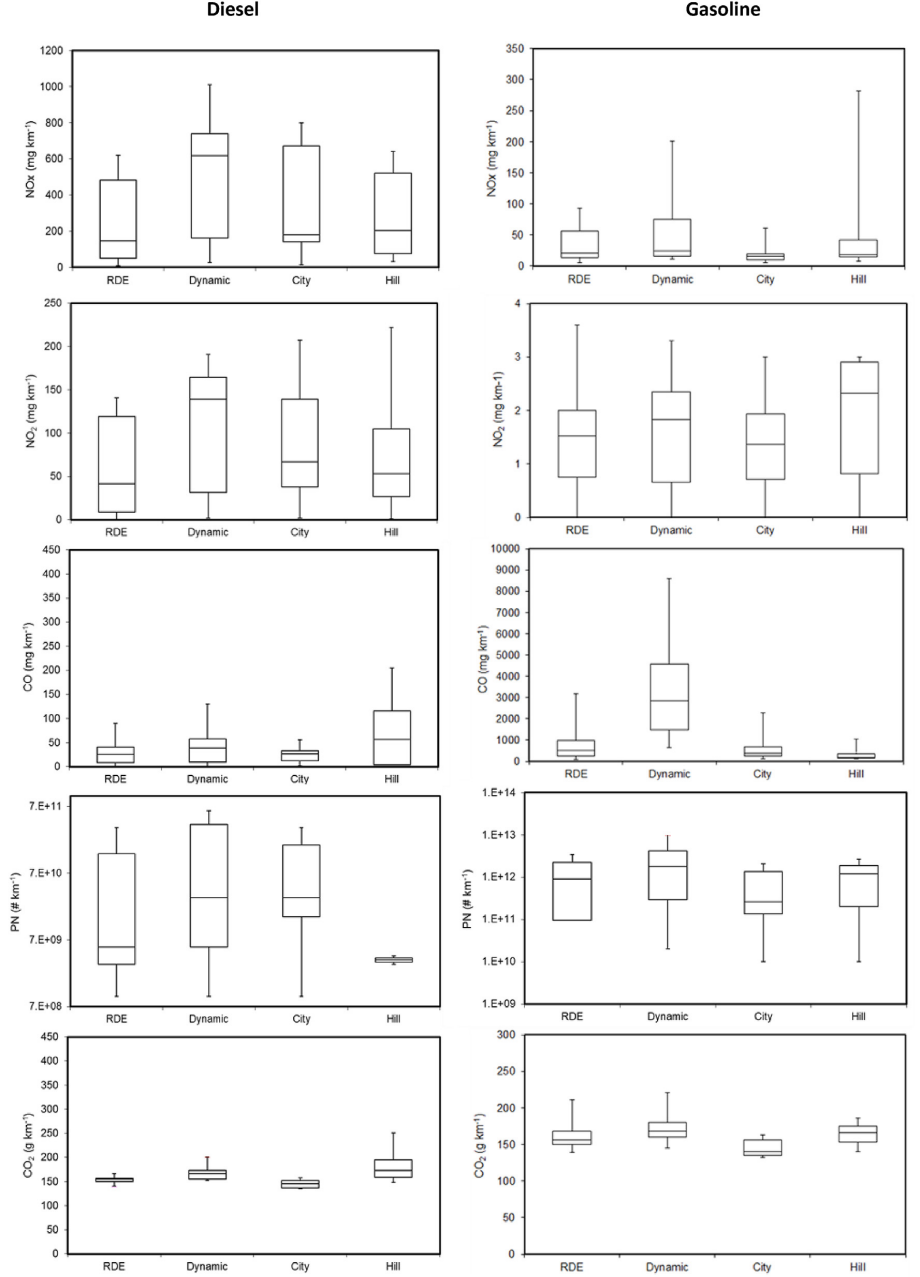
NOx, NO_2_, CO, PN and CO_2_ emission factors from pre-RDE vehicles, Euro 6b diesel vehicles (DV1-DV7) and Euro 6b and 6c light-duty gasoline vehicles (GV1-GV8) vehicles, during the RDE, Dynamic, City-Motorway (City) and Hill routes. These vehicles were type approved under the NEDC (i.e., not RDE requirements). For the calculation of the RDE and Dynamic tests the individual emissions of the two routes (RDE-1 and RDE-2) were used. The middle bar indicates the median, the bottom indicates the first quartile, the top the third quartile and the ends of the whisker are set at 1.5*IQR (interquartile range) above the third quartile and 1.5*IQR below the first quartile.

**Fig. 2:**
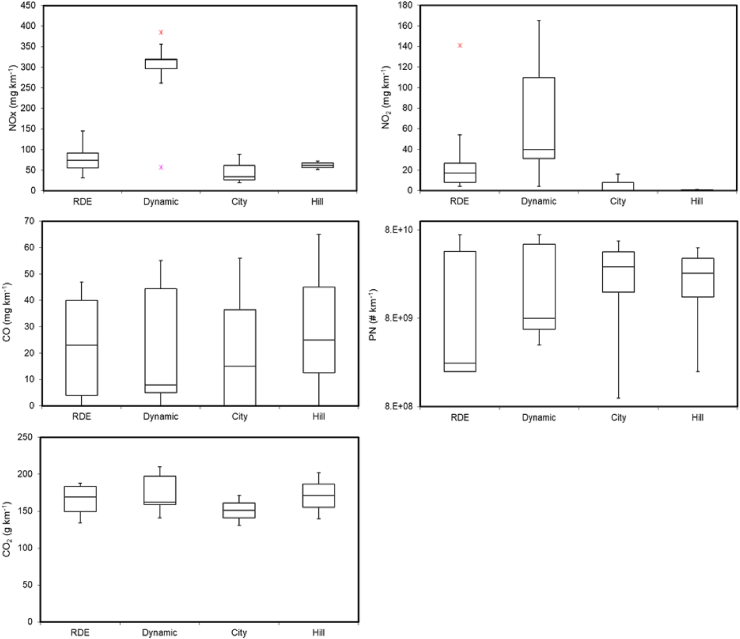
NOx, NO_2_, CO, PN and CO_2_ emission factors for Euro 6d-TEMP light-duty diesel vehicles (DV8-DV10) vehicles (all type approved under WLTP and RDE) during the RDE, Dynamic, City-Motorway (City) and Hill routes. For the calculation of the RDE and Dynamic tests the individual emissions of the two routes (RDE-1 and RDE-2) were used. The middle bar indicates the median, the bottom indicates the first quartile, the top the third quartile and the ends of the whisker are set at 1.5*IQR (interquartile range) above the third quartile and 1.5*IQR below the first quartile. Asterisks represent statistical outliers.

Fig. 1 and Fig. 2 illustrate NOx, NO_2_, CO, PN and CO_2_ median emissions factors from diesel and gasoline vehicles obtained during the tests performed using the different routes and driving styles. Table 5 summarizes the CO, NO_2_, NOx, CO_2_ and PN emission factors for each individual vehicle. The emissions factors indicated as RDE and Dynamic are the mean of the emissions obtained using routes RDE-1 and RDE-2 for the RDE compliant tests and the dynamic tests, respectively.

**Table 5:** Emission factors of NOx, NO_2_, CO, PN ( × 10^11^ #/km) and CO_2_ during the RDE, Dynamic (Dyn.), City-Motorway (City-MW) and Hill tests and routes. The emissions factors presented in the columns RDE and Dyn. are the average of the emissions obtained along the two different routes (RDE-1 and RDE-2) tested using these two different driving dynamics (RDE and Dynamic).

	NOx mg/km				NO_2_ mg/km				CO mg/km				PN ( × 10^11^ #/km)				CO_2_ (g/km)			
Code	RDE	Dyn.	City-MW	Hill	RDE	Dyn.	City-MW	Hill	RDE	Dyn.	City-MW	Hill	RDE	Dyn.	City-MW	Hill	RDE	Dyn.	City-MW	Hill
GV1	6	13	9	8	0	0	0	0	681	2829	370	1046	6	11	2.6	10	155	167	163	140
GV2	15	12	11	19	3	3	3	3	2192	6234	2234	450	2	4.5	1.7	5.1	156	152	135	166
GV3	39	33	21	56	1	0	0	3	208	1228	455	167	12	28	8.9	19	129	149	137	156
GV4	91	93	62	288	2	3	2	2	990	2577	930	199	24	34	21	27	140	164	132	167
GV5	11	20	5	10	1	2	1	1	317	864	108	115	0.9	1.2	1	1	154	168	140	142
GV6	21	16	–	–	0	0	-	-	988	7551	–	–	21	104	–	–	201	215	–	–
GV7	46	92	18	25	1	2	-	-	433	3303	307	127	31	62	18	19	166	180	154	186
GV8	20	115	16	18	2	7	2	3	161	2737	181	149	0.2	0.3	0.1	0.1	172	184	158	182
DV1	476	702	–	639	131	176	–	236	334	243	–	205	–	–	–	–	188	203	–	381
DV2	552	784	799	405	124	125	207	97	25	25	32	129	–	–	–	–	139	154	145	155
DV3	569	1005	673	641	127	190	139	113	25	75	34	103	0.03	0.01	<0.01	0.04	155	165	158	148
DV4	147	645	179	203	42	157	38	53	5	6	9	4	–	–	–	–	158	173	152	173
DV5	33	85	–	59	7	10	–	16	35	36	–	57	0.17	2	–	0.15	156	177	–	194
DV6	78	300	141	93	22	104	67	38	13	30	21	4	5	6.1	3.4	6.4	150	162	137	197
DV7	13	39	13	31	1	2	2	1	20	27	30	65	0.06	0.2	0.3	0.03	152	153	135	162
DV8	60	188	19	72	6	14	0	0	20	31	0	25	0.7	0.7	0.6	0.5	139	150	131	140
DV9	57	319	34	51	13	40	0	1	4	0	0	0	0.02	0.06	<0.01	0.02	188	209	171	202
DV10	119	338	89	–	27	110	16	–	35	24	56	–	0.03	0.07	0.01	–	169	162	151	–
CNG-LCV	164	956	242	515	15	33	16	21	307	208	440	317	6	–	–	–	243	282	251	268

Median NOx emissions from gasoline vehicles ranged from 16 mg/km during the City-Motorway test to 21 and 24 mg/km during the RDE and dynamic tests, respectively. The lowest NOx emissions resulted from the GV1 and GV5 (5 mg/km) in the RDE and City-Motorway tests respectively and the highest from the GV4 during the Hill test (288 mg/km). The emission factors were in general low. While NOx emissions during dynamic tests were similar or lower than RDE for half of the tested vehicles (vehicles GV2, GV3, GV4 and GV6), they were more than twice higher for the other half (vehicles GV1, GV5, GV7 and GV8; see Table 5). These higher emissions took place during the urban and rural sections of the on-road trips (see Table S1 of the supplementary material).

Median NOx emission factors obtained during the on-road tests were in excellent agreement with the median NOx emission factor obtained for the gasoline vehicles derived from WLTP tests conducted at 23 °C (24 mg/km) (Clairotte et al., 2018). They were also in excellent agreement with the average NOx reported for twelve Euro 6b gasoline vehicles tested in the laboratory under the WLTP at 23 °C (22 mg/km) (Suarez-Bertoa and Astorga et al. 2018). This indicates that gasoline NOx emission control technology (three-way catalyst) exhibits a consistently good performance, both in laboratory and in real-world conditions.

Most gasoline vehicles complied with the NOx Euro 6d-TEMP on-road NTE (i.e., M1 Class, 60 mg/km multiplied by a conformity factor of 2.1) with the exception of GV8 during one of the dynamic tests (205 mg/km) and the GV4 during the Hill route (288 mg/km). In addition, NOx emissions higher than the 6d limits (M1 Class, 60 mg/km multiplied by a conformity factor 1.43) were measured from GV7 during the Dynamic test (92 mg/km) and GV4 during RDE and Dynamic (89 and 93 mg/km respectively).

NOx median emissions factors from the pre-RDE diesel vehicles fleet (DV1 – DV7) were one order of magnitude higher than those of the gasoline fleet, and varied from 147 mg/km, during the RDE compliant tests, to 617 mg/km during the Dynamic tests. Individual average emission factors ranged 9 mg/km (DV7) during the RDE test to 1011 mg/km (DV3) during the dynamic tests. The Euro 6b diesel vehicles DV1 – DV4 (type approved under the NEDC) tested under RDE compliant tests presented NOx emissions from similar (DV4) to up to 4 times higher than the RDE Euro 6d-TEMP limit for diesels (i.e., M1 Class, 80 mg/km multiplied by a conformity factor of 2.1) and 1.4 to 6 times higher than the RDE Euro 6d standard (i.e., M1 Class, 80 mg/km multiplied by a conformity factor of 1.43). Vehicle DV4 met the Euro 6d-TEMP tailpipe emissions requirements (but fell short of meeting Euro 6d). Vehicles DV5 and DV6 met the more stringent RDE Euro 6d. The elevated NOx emissions from Euro 6b vehicles has been explained by the after-treatment strategies and low efficiency of the catalytic systems used in those vehicles to reduce the emissions of NOx, namely SCR or LNT (Ko et al., 2017; O'Driscoll et al., 2016, 2018; Suarez-Bertoa et al., 2015b; 2016a; 2017; Yang et al., 2015).

NOx median emissions factors from the Euro 6d-TEMP diesel vehicles fleet (DV8 – DV10) ranged from 34 mg/km during the City route, to 318 mg/km during the Dynamic tests. These vehicles were type approved to the Euro 6d-TEMP standard using WLTP and RDE tests. On the other hand, an RDE test was not required for vehicles DV5, DV6 and DV7 at the time of type approval. Nonetheless, together with the Euro 6d-TEMP vehicles (DV8 – DV10), they met the Euro 6d NOx tailpipe emissions requirements during the RDE, City and Hill tests. Only during the Dynamic tests were the NOx emission factors higher than Euro 6 limits. Vehicle DV7 managed very low emissions for this test (40 mg/km). The increase of NOx emissions with dynamicity (as measured in terms of v*a) is in good agreement with the findings of Gallus et al., (2017) for a Euro 5 and a Euro 6b diesel vehicle. This indicates that although there is room for improvement, substantial progress has been made on NOx emission control in the more recent, Euro 6d-TEMP vehicles, and that high reduction efficiency of NOx is often maintained beyond the dynamic boundaries of RDE. Vehicles DV7, DV8 and DV9 achieved lower NOx emissions compared to the Euro 6b diesel vehicles by using more advance and complex catalytic systems (e.g., EGR + LNT + SCR, EGR + dual-LNT) and, possibly, a higher urea solution dosage in the SCR. Interestingly, NOx emissions from the Euro 6d-TEMP vehicles were low also during the second urban section of the City-MW route including the low-load operation (Fig. S1 of the supplementary material). We recently reported that a Euro VI diesel heavy-duty vehicle equipped with a second generation SCR tested using the same route (Mendoza-Villafuerte et al., 2017), presented high NOx emissions during low-load operation, even when the SCR had reached operation temperature conditions. This indicates that the tested Euro 6d-TEMP diesel light-duty vehicles use a more suitable system, or set of systems, for urban operation than those used in the tested Euro VI diesel heavy duty.

NOx emissions from the CNG-LCV were the highest recorded among the positive ignition vehicles (see Table 5). They ranged from 164 mg/km during the RDE route to 956 mg/km during the Dynamic routes, which is 9 times higher than the worst performing gasoline vehicle. It should be noted that this vehicle (CNG-LCV) was a light commercial vehicle whereas the other tested vehicles were passenger cars. Surprisingly, NOx emissions from the CNG-LCV were comparable to or higher than most of the diesel vehicles measured in this study. The high NOx emissions may be linked to lean engine operation, i.e., an engine operation that uses a higher air to fuel ratio (lambda >1) than the stoichiometric (lambda = 1). More air in the combustion chamber lead to higher engine-out NOx emissions. The results obtained during the RDE tests are in very good agreement with those reported by Vojtisek-Lom et al., (2018) for a dual CNG-Gasoline LCV tested on-road (186 mg/km).

Ambient temperature has been shown to affect NOx emissions from passenger cars (Kwon et al., 2017). However, in the current study, where temperature varied from 3 °C to 33 °C, no particular trend was observed for the studied fleet (Fig. S2 of the supplementary material). Furthermore, since each vehicle was tested during the same period of the year, there are not enough data to analyse the inter-vehicle variability related to ambient temperature variations. Moreover, no particular trend was observed for CO or PN from the tested gasoline vehicles (Fig. S2 of the supplementary material).

Before the introduction of DPF and SCR systems, NOx in diesel exhaust was usually composed of >90% NO. However, to decrease soot oxidation temperatures for the DPF regeneration and since equimolar amounts of NO and NO_2_ increase the reaction rate with NH_3_ on the SCR, NO is oxidised to NO_2_ on the DOC (Guan et al., 2014).

NOx emissions from the spark ignition vehicles tested were mainly composed of NO. Median NO_2_ emissions from gasoline cars were very low (about 2 mg/km during all the tested routes with a maximum of 4 mg/km). On the other hand, median NO_2_ emissions from pre-RDE diesel vehicles (DV1-DV7) ranged from 42 to 139 mg/km, during the RDE and Dynamic routes respectively. In the case of DV8-DV10 (Euro 6d-TEMP vehicles) median NO_2_ emissions range from below limit of detection during City-MW route to 40 mg/km during Dynamic route. Hence, Euro 6d-TEMP vehicles, mainly those equipped with SCR, presented lower NO_2_ emissions factors than pre-RDE vehicles during all the studied conditions (see Table 5). Vehicles DV7 and DV8 emitted less than 5% of NOx as NO_2_. Vehicles DV9 and DV10 had NO_2_:NOx ratios (0.2) similar to those from Euro 6b vehicles.

The results obtained for pre-RDE vehicles are in good agreement with recent studies that have reported an increase on the NO_2_:NO ratio in the modern diesel cars exhaust (O'Driscoll et al., 2016; Suarez-Bertoa and Astorga, 2018 Suarez-Bertoa et al., 2019). The higher ratio of NO_2_ emissions in the exhaust may have important effects on the urban atmospheric chemistry, and consequently on air quality. The EEA has recently reported that, following an increase of NO_2_ emissions from diesel vehicles at the expense of NO, ground level ozone (O_3_) concentrations have increased in several air quality measurement stations monitoring pollution from traffic in the EU (European Environmental Agency, 2018). Hence, further studies will be needed to evaluate the impact that relatively low vehicular NO and NO_2_ emissions with high NO_2_:NO ratios, as those seen for SCR-equipped Euro 6d-TEMP vehicles, will have on urban O_3_.

PN emissions from diesel vehicles were below Euro 6 limits (6 × 10^11^ #/km) under all the studied conditions for all the studied vehicles even without applying the applicable conformity factor of 1.5 for PN. PN median emissions ranged from 6 × 10^9^ #/km to 3 × 10^10^ #/km for both, pre-RDE and Euro 6d-TEMP vehicles. The measured PN emissions indicate a general good performance of DPFs during real-world operation. Due to the very low emissions and the high variability there was no significant difference on PN emissions for the different routes used (see Fig. 1). The results were comparable to those reported in previous laboratory and on-road studies for Euro 6b vehicles (Giechaskiel et al., 2015; Suarez-Bertoa and Astorga, 2018; Suarez-Bertoa et al., 2019).

Some of the diesel vehicles regenerated during their on-road testing, but nevertheless, the emissions remained <6 × 10^11^ #/km. Generally, the PN levels of diesel vehicles remain below the limit of 6 × 10^11^ #/km even when the emissions during regeneration events are considered, as the evaluation of Giechaskiel et al. (2018a, 2018b) showed. The levels remain still below the laboratory PN limit of 6 × 10^11^ #/km, in agreement with the observations of Giechaskiel et al., (2018a). In that study it was shown that the contribution of the emissions during regeneration could be from negligible up to significant. In any case, it is important to consider the emissions during regeneration emissions in the next regulatory step for light-duty vehicles.

PN emissions from gasoline vehicles were up to three orders of magnitude higher than those obtained from diesel vehicles. Median PN emissions from gasoline vehicles varied from 3 × 10^11^ #/km to 2 × 10^12^ #/km. They are below the laboratory PN limit for GDIs until 2017 (6 × 10^12^ #/km), even without any additional margin. PN emissions were comparable to those reported in previous on-road (Ko et al., 2019; Demuynck et al., 2017; Joshi and Johnson, 2018; Giechaskiel et al., 2015) and laboratory studies for both GDI and PFI gasoline vehicles without a GPF (Braisher et al., 2010; Chan et al., 2013; Zhu et al., 2016; Suarez-Bertoa and Astorga, 2018). PN emissions from GDIs are higher due to the limited time available for fuel and air to be thoroughly mixed in the combustion chamber; these emissions increase during high-speed and sudden acceleration events due to rich air/fuel ratios (Überall et al., 2015; Yinhui et al., 2016). On the other hand, PN emissions from PFI spark ignition vehicles are commonly linked to enrichment of the air-fuel mixture during cold start engine operations and accelerations. Although most of the GDIs studied here resulted in higher PN emissions than the PFIs, PN emissions from the PFIs exceed in some occasions the PN limits for diesel and GDI vehicles. High emissions of PFIs especially at dynamic cycles is not new; similar levels were found for Euro 1–3 (Ntziachristos et al., 2004), but even for recent Euro 5–6 vehicles (Hensel et al., 2018; Giechaskiel et al., 2018a). It needs to be seen if future GPFs will be able to reach the emission levels of efficient DPFs (one order of magnitude lower).

**Fig. 3:**
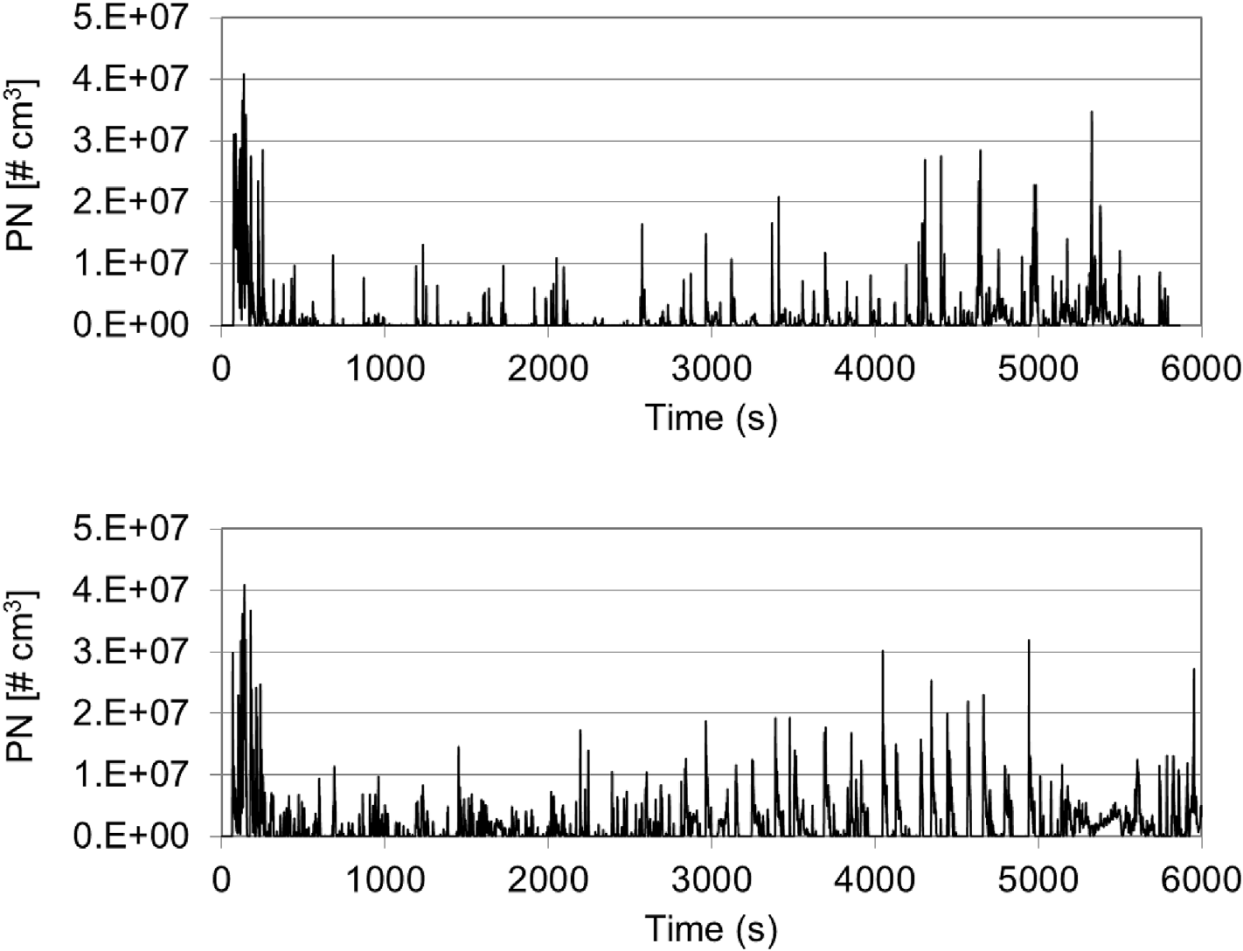
PN emission profiles from GV6 during the RDE tests along RDE-1 (top) and RDE-2 (bottom) routes.

The highest PN emissions were recorded for vehicle GV6, a PFI gasoline car, which not only exhibited very high PN emissions during the cold-start phase, but also across all trip sections (see Fig. 3). Emissions ranged from 2 × 10^12^ #/km during the RDE routes to 1 × 10^13^ #/km during the dynamic routes. The lowest PN emissions resulted from vehicle GV8 (1 × 10^10^–2 × 10^10^ #/km), a GDI vehicle equipped with a gasoline particle filter (GPF). PN emissions from GV8 were approximately one order of magnitude lower than those reported for GPF retrofitted GDIs during on-road tests (Ko et al., 2019; Demuynck et al., 2017). Although this was the only gasoline vehicle equipped with a GPF, the consistency of the results with those from previous studies indicate that GDIs equipped with a GPF consistently achieve much lower PN emissions than those without the GPF.

PN median emissions were unsurprisingly higher – 2 times– during the dynamic routes (2 × 10^12^ #/km) than during the RDE routes (1 × 10^12^ #/km). More dynamicity means more accelerations episodes, which in turn results in richer the air/fuel ratios, hence in higher PN emissions. PN median emissions were 3.5 times lower during the City-Motorway route than during the RDE ones. If the GV6, the highest emitter and which was not tested along this route, is excluded from the analysis, PN median emissions result 2.4 times lower during the City-Motorway route than during the RDE. In any case, as illustrated in Fig. 1, there were no significant differences on PN emissions for different routes. PFIs though had tendency for higher emissions during the Hill route.

Due to an instrument failure, PN emissions from the CNG-LCV vehicle were only measured during the RDE tests. PN emissions were as high as those recorded from GDI vehicles reaching 1 × 10^12^ #/km during the RDE-1 route. PN average emission factor during the RDE routes was 6 × 10^11^ #/km. These results are in line with those reported for a dual CNG-gasoline tested using the US06 and Artemis cycles (Vojtíšek-Lom et al., 2018), both more dynamic and broadly considered to be more representative of real-world driving than NEDC. It should be noted that the specific vehicle had <3000 km during the on road testing, so the contribution of fresh lubricant could be significant.

This study did not evaluate the solid particle emissions below 23 nm, which could be high for gasoline vehicles exceeding +50% of the >23 nm emissions (Giechaskiel et al., 2018a) and even higher for CNG vehicles (Giechaskiel et al., 2019). Including particles below 23 nm and keeping the emission limit the same could result in GV1 and CNG-LCV exceeding the limit.

The current regulation does not include volatile particles because the main target of the regulation was to force the best available technology (DPFs) with good repeatability and reproducibility (Giechaskiel et al., 2012). However, there were concerns that reduction of soot particles could result in increase of nucleation mode volatile particles (Vouitsis et al., 2004; Du and Yu 2008). In addition, regeneration increases significantly the nucleation mode particles (Dwyer et al., 2010; Mamakos et al., 2013). The importance of volatile particles is a topic of research. However, in order to introduce it in the regulation with good repeatability and reproducibility sampling from the tailpipe with fixed parameters will be necessary (Keskinen and Rönkkö 2010).

CO emissions from diesel vehicles were below Euro 6 limits (500 mg/km) under all the studied conditions for all the studied vehicles. CO median emissions range between 26 and 57 mg/km for pre-RDE vehicles (DV1-DV7) and between 8 and 25 mg/km for Euro 6d-TEMP vehicles (DV8-DV10). There was no significant difference on CO emissions for the different routes used (see Figs. 1 and 2). The results were comparable to those reported in previous laboratory and on-road studies for Euro 6b vehicles (Suarez-Bertoa and Astorga, 2018; Suarez-Bertoa et al., 2019). CO median emissions were one order of magnitude lower than those reported by Costagliola et al., (2018) for two Euro 5b diesel vehicles tested on-road, and whose emissions (150–370 mg/km) were however comparable to DV1 and DV3, which presented the highest CO emissions of all of our tests. The measured CO emissions indicate low engine-out CO emissions and/or good operation of the Diesel oxidation catalysts (DOCs).

CO median emissions from gasoline vehicles were one to two orders of magnitude higher than those obtained from diesel vehicles. Median CO emissions from gasoline vehicles ranged from 167 mg/km during the Hill test to 2850 mg/km during the dynamic test (see Fig. 1). CO emissions during the RDE, City-Motorway and Hill routes were comparable to those previously reported for Euro 6b gasoline vehicles tested over an RDE-like chassis dynamometer test cycle (Demuynck et al., 2017) and over WLTP at 23 °C (Suarez-Bertoa and Astorga, 2018). However, CO emissions during dynamic tests were more than five times higher than those obtained during the RDE compliant tests. A similar trend, of increasing CO emissions with stronger dynamics was also reported by Demuynck et al., (2017) during on-dyno RDE tests. Previous studies have shown that high CO emissions from gasoline cars resulting from rich engine operation are often accompanied by emissions of NH_3_ after the light-off of the three-way catalyst (Livingston et al., 2009; Kean et al., 2009; Suarez-Bertoa et al., 2014; 2016a). NH_3_ is one of the main atmospheric precursors of PM_2.5_ and promote the formation of secondary organic aerosols (Link et al., 2017; Kirkby et al., 2011). Recent studies have shown that measurement of on-road emissions of NH_3_ is possible using FTIR and QCL-IR (Vojtíšek-Lom et al., 2018, Suarez-Bertoa et al., 2017; 2016b). This will allow the measurement of NH_3_ emissions in future testing campaigns.

**Fig. 4:**
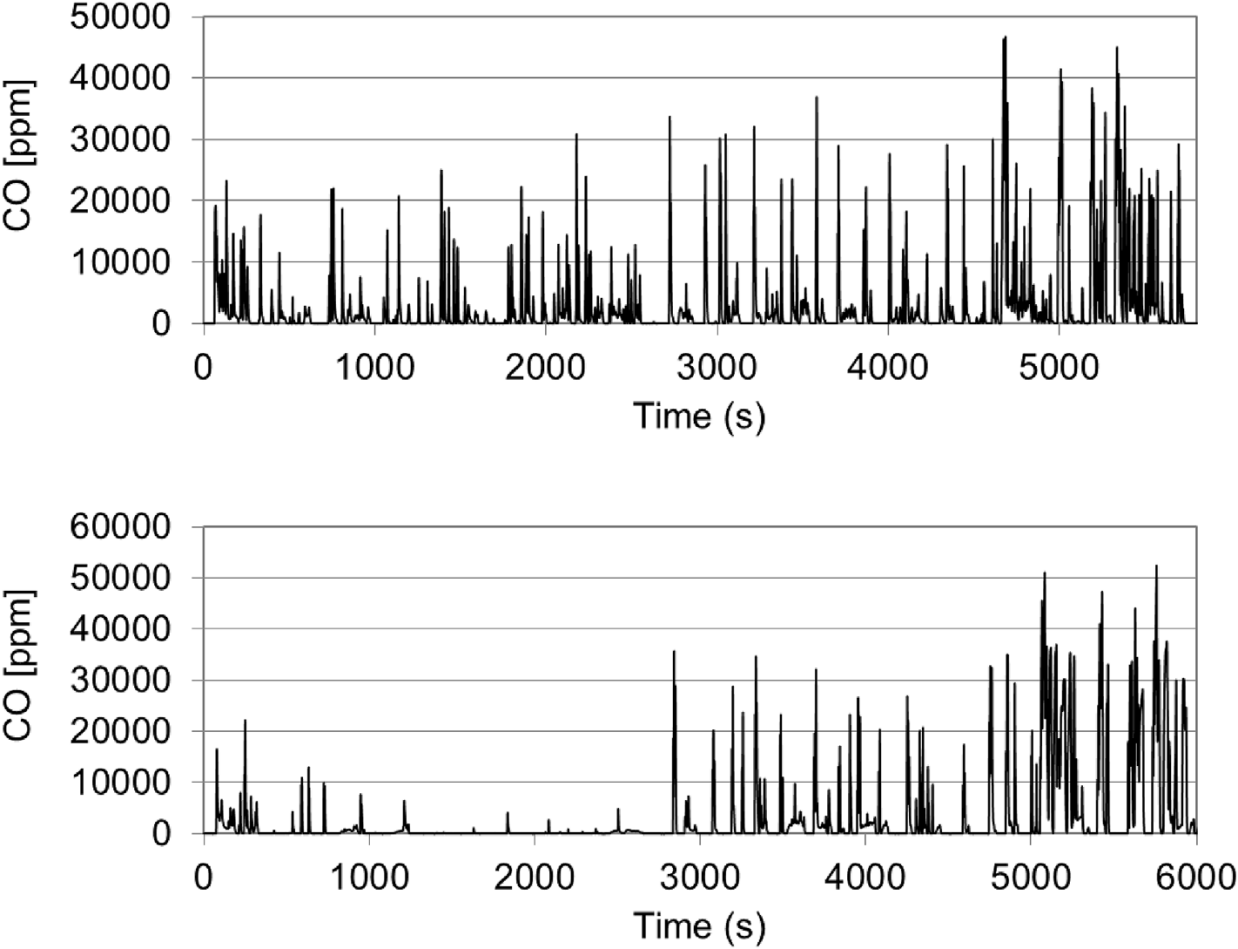
CO emission profiles from GV6 (top) and GV2 (bottom) during a dynamic test along RDE-1 route.

Very high CO emissions were recorded for most gasoline vehicles during Dynamic trips, reaching concerning levels of 6000–7500 mg/km (see in particular GV2 and GV6); approximately 8 times more compared to the non-dynamic driving. As illustrated in Fig. 4, these high CO emissions may take place during any trip section during dynamic driving. For some vehicles they were associated to motorway operation during dynamic tests as well as RDE compliant test (see Fig. S3 in the supplementary material). These may be a consequence of an emissions strategy (AES) aiming to protect the TWC from overheating, but also due to an undersized catalyst. Since engine-out emissions were not measured during the testing campaign, it was not possible to examine the behaviour of the catalyst during these emission events, but further investigations will be conducted in future testing campaigns.

Regardless of its large engine displacement and high laden mass, the CNG-LCV exhibited the lowest CO emissions of all the positive ignition vehicles during the Dynamic tests, 208 mg/km. Nonetheless, CO emissions for this vehicle during the City-Motorway test (440 mg/km), were in agreement with the median of the other vehicles tested during City-Motorway (416 mg/km).

The PEMS units used during this study did not include a flame ionization detector (FID) module. Hence, it was not possible to assess the emissions of hydrocarbons, THC or CH_4_, during the on-road tests. Nonetheless, in view of the CO and PN emissions measured from the gasoline and the CNG vehicles, the emissions of THC and CH_4_ should be addressed in future studies.

Median CO_2_ emissions from diesel vehicles were slightly higher than median CO_2_ emissions from the gasoline vehicles, contrary to normal expectation. Diesel cars are also more prevalent in heavier, larger segments (e.g. SUVs). Indeed, the vehicles tested presented in this study included several relatively small gasoline engines with average engine displacement of ~1150 cc and average power of ~75 kW. On the other hand, the average engine displacement and power of the diesel vehicles tested were ~1900 cc and ~112 kW. In addition, 5 out of 8 gasoline vehicles tested were GDIs, which are generally more efficient than PFIs.

Median CO_2_ emissions increased for Dynamic trips in relation to RDE-compliant trip (+7% and +6% for gasoline and diesel, respectively). The highest impact of dynamic driving was measured for the CNG vehicle (+16%) which was also the heaviest vehicle. The City-Motorway driving resulted in lower CO_2_ emissions compared to the RDE-compliant routes (−6% for both gasoline and diesel vehicles). The most energy-demanding route for diesel vehicles was the Hill route which on average resulted in 10% higher CO_2_ emissions. In particular, this route was the most demanding for vehicles DV5 and DV6 (25% and 29% increase in CO_2_ emissions, respectively). For gasoline vehicles, the Hill route exhibited 6% higher CO_2_ emissions (average of all vehicles), with some gasolines vehicles (GV1 and GV5) achieving lower CO_2_ emissions compared to RDE-compliant trips. It should be noticed that the Hill route begins and ends at the same point. Therefore, CO_2_ emissions are the combination of and uphill (Max. altitude ~1100m.a.s.l.) and downhill driving (Min. altitude ~200m.a.s.l.). CO_2_ emission factors (g/km) were up to ~120% higher when driving uphill than downhill in agreement with a previous study (Giechaskiel et al., 2018b). CO_2_ emission factors during the uphill driving segment were 40–80% higher than during the tests compliant with RDE ambient, trip and dynamic boundary conditions (RDE).

The three vehicles (DV8-DV10) analyzed in the present study were type-approved following the new provisions of the WLTP testing procedure (Euro 6d-temp vehicles) and therefore had CO_2_ emission results (available in the Certificate of Conformity) for both NEDC and WLTP tests. The average CO_2_ emissions measured for these vehicles from RDE and non-RDE compliant routes were higher by 13% and 11% (respectively) compared to the WLTP declared values and 44% and 43% (respectively) higher compared to the NEDC declared CO_2_ emissions. These results confirm the findings of the previous study that, for what concerns the CO_2_ emissions and fuel consumption, indicates that the WLTP results in an improvement compared to the NEDC and it is able to significantly reduce the difference between the type approval and RDE CO_2_ emissions (Pavlovic et al., 2018).

## 4 Conclusions

The on-road emissions of NOx, NO_2_, CO, PN and CO_2_ from nineteen Euro 6 vehicles, including diesel (three of which were type-approved to the recent Euro 6d-TEMP standard) gasoline (GDI and PFI) and CNG vehicles, were investigated over two RDE-compliant routes and other tests designed to explore driving situations outside the boundary conditions of RDE (dynamic driving, different shares of urban/rural/motorway operation, uphill driving).

Our results indicate that, following the introduction of new vehicle emissions test procedures in EU – WLTP and RDE– more efficient and complex NOx emission control technologies (both in terms of hardware and software) are being used in diesel vehicles. Consequently, these diesel vehicles exhibit markedly lower NOx emissions than earlier Euro 6 diesel vehicles for the RDE-compliant tests and for some of the more demanding tests outside RDE boundary conditions. This is an encouraging sign of the ability of RDE-compliant vehicles (Euro 6d-TEMP and later) to deliver consistently low-NOx emission performance. Nonetheless, substantially increased emissions during the Dynamic tests (which were also recorded for some of the gasoline cars) indicate there is room for improvement.

The measured PN and CO emissions from all diesel vehicles also point to a very good performance of DPFs and DOCs during real-world operation. However, pollutant emissions that were not a matter of concern at the time the Euro 6 standards were developed, such as CO emissions from gasoline vehicles, or PN emissions from PFI gasoline vehicles, were shown to be very high in some instances. Our results, although based on a limited sample, indicate that gasoline vehicles can - under certain conditions - exhibit substantial emissions of CO during RDE tests, and these can be up to eight times higher for Dynamic tests. Moreover, not only GDI vehicles exhibited high PN emissions: PN emissions, sometimes >6 × 10^11^ #/km, were also recorded for PFI vehicles (for which there is no PN emission limit): one vehicle during normal RDE and two during dynamic RDE. On the other hand, the only GPF-equipped vehicle exhibited the lowest PN emissions of the gasoline vehicles by far (more than one order of magnitude below the PN limit).

Our work points to the relevance of a technology- and fuel-neutral approach to vehicle emission standards, whereby all vehicles must comply with the same emission limits for all pollutants. In light of the findings of our study (although based on a limited sample of gasoline vehicles) indicating the increase of CO emissions during dynamic operation, more work on this topic is advised. Finally, it is worth noting that several boundary conditions of RDE were not explored in our work (notably cold temperatures, dense traffic, and driving at high altitude). These will be the subject of further research.

## Disclaimer

The opinions expressed in this manuscript are those of the authors and should not be considered to represent an official position of the European Commission.

Mention of trade names or commercial products does not constitute endorsement or recommendation by the authors or the European Commission.
