# Supplementary material for: On-road emissions of passenger cars beyond the boundary conditions of the real-driving emissions test
Source: Environ Res. 2019 Sep;176:108572. doi: 10.1016/j.envres.2019.108572 (PMC6722398; doi:10.1016/j.envres.2019.108572)
Supplement: Multimedia component 1 [file mmc1.docx]

**On-road emissions of passenger cars beyond the boundary conditions of the Real-Driving Emissions test.**

Suarez-Bertoa Ricardo,^*1^ Valverde Victor,^1^ Clairotte Michael,^1^ Pavlovic Jelica,^1^ Giechaskiel Barouch,^1^ Franco Vicente,^2^ Kregar Zlatko,^2^ and Astorga Covadonga.^*1^

*^1^European Commission Joint Research Centre (JRC), Ispra, Italy.*

*^2^European Commission Directorate-General for Environment, Brussels, Belgium.*

*Corresponding authors.

E-mail addresses: ricardo.suarez-bertoa@ec.europa.eu (Suarez-Bertoa R.);

covadonga.astorga-llorens@ec.europa.eu (Astorga C.)

T1. Specific requirements for N2 vehicles during RDE and emission limits for Euro 6 positive ignition N2 vehicles.

i) For N2 category vehicles that are equipped in accordance with Directive 92/6/EEC with a device limiting vehicle speed to 90 km/h:

 - rural operation speed > 60 km/h and <= 80 km/h.

  - motorway operation speed > 80 km/h.

  - speed range of the motorway driving of shall properly cover a range between 80 and 90 km/h.

  - motorway speed above 80 km/h for at least 5 minutes.

Moving Average Window:

  - the share of motorway windows in the complete test shall be at least 5 %.

ii) Upon the request of the manufacturer, and only for those N1 or N2 vehicles where the vehicle power-to-mass ratio is less than or equal to 44 W/kg then:

If vk ≤ 74,6km/h and 〖(v∙a_pos)〗_(k) [95] > (0,136∙vk+14,44)  is fulfilled, the trip is invalid.

If vk > 74,6km/h and (〖v∙a〗_pos )_k_[95] > (-0,097∙vk  + 31,635) is fulfilled, the trip is invalid.

To calculate the power-to-mass ratio, the following values shall be used:

- the mass which corresponds to the actual test mass of the vehicle including the drivers and the PEMS equipment (kg);

- the maximum rated engine power as declared by the manufacturer (W).

Figure S2. NOx emission profiles for DV8 (top), DV9 (centre) and DV10 (bottom) during the City-MW trips.

DV8


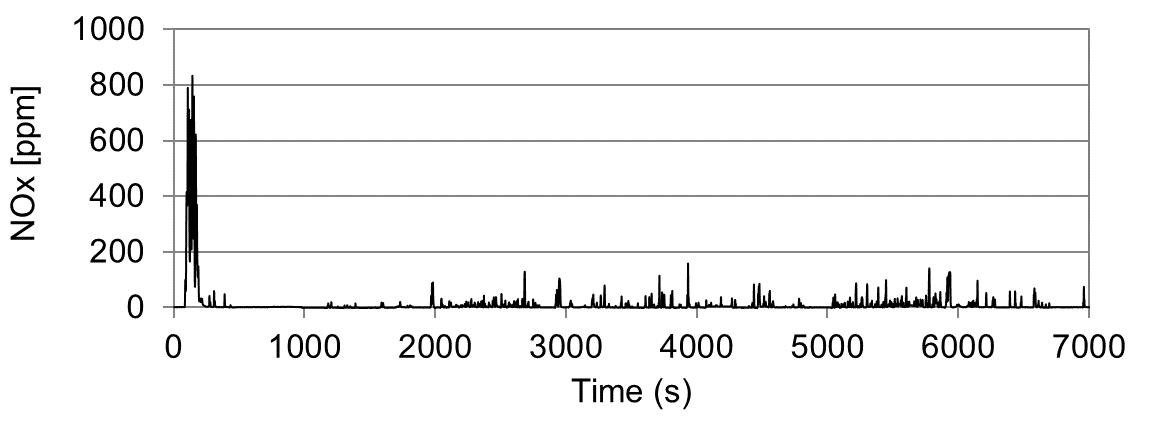


DV9


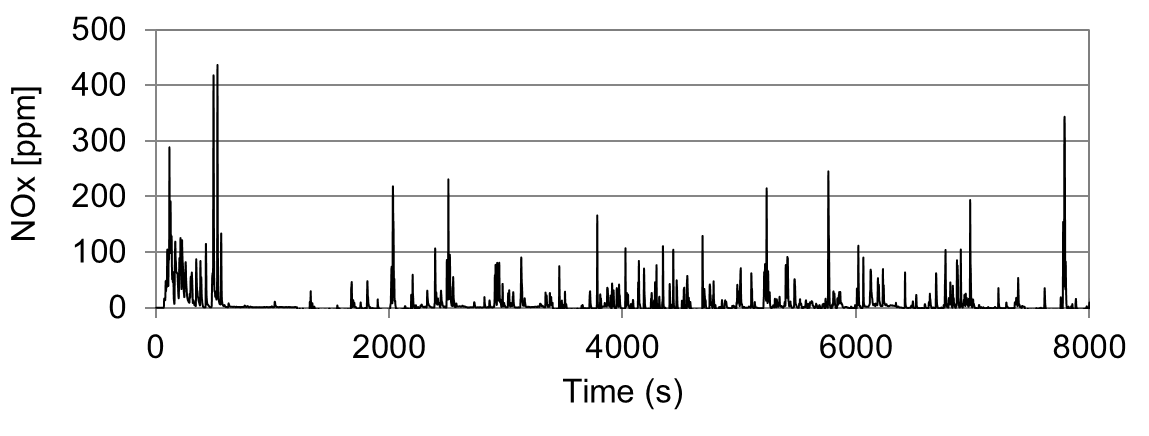


DV10


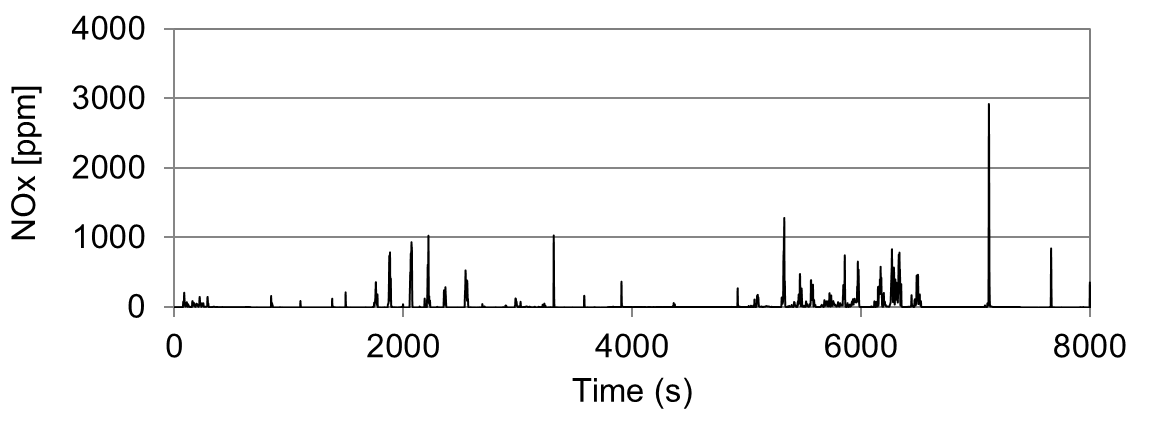


Figure S2. Emissions of NOx, CO and PN *vs* the average ambient temperature during the tests.


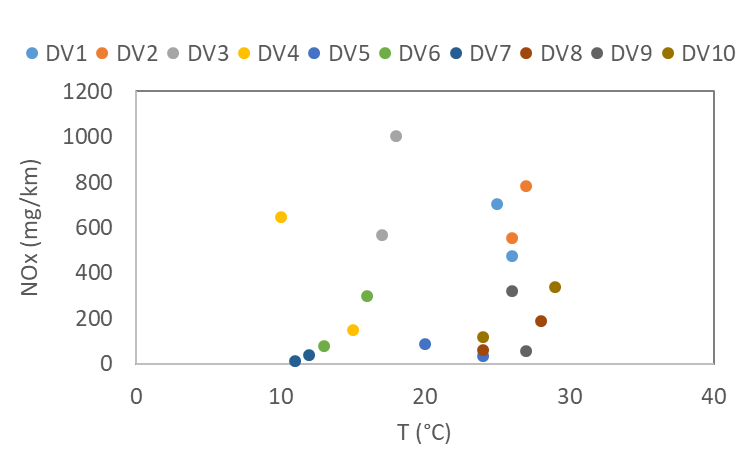

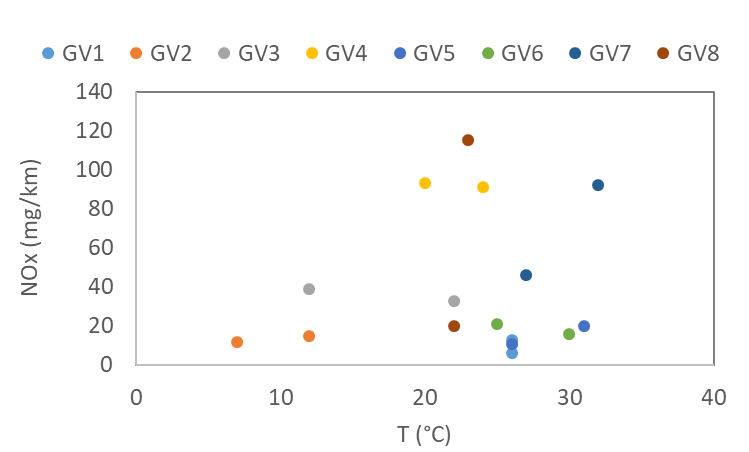


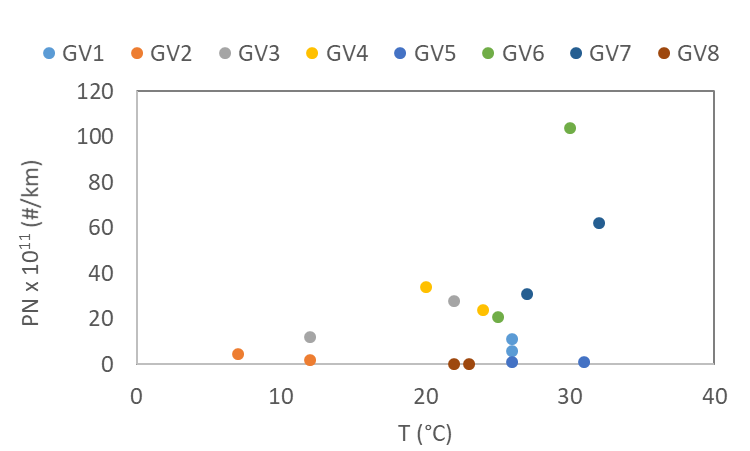

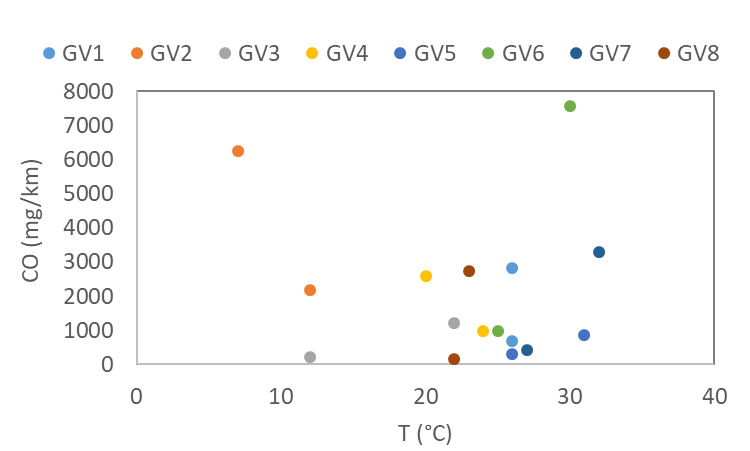


Figure S3. CO emission profiles from GV2 during an RDE compliant test (top) and a dynamic test (bottom) along RDE-2 route.


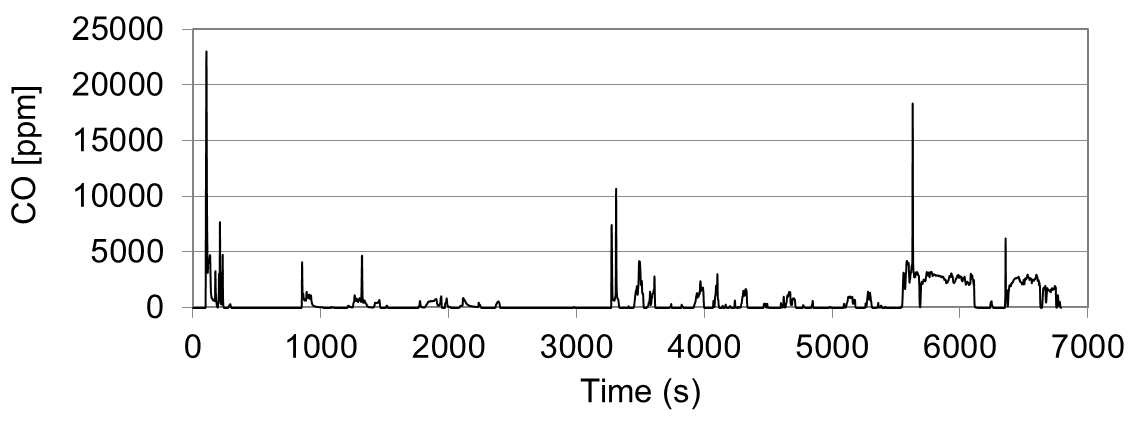


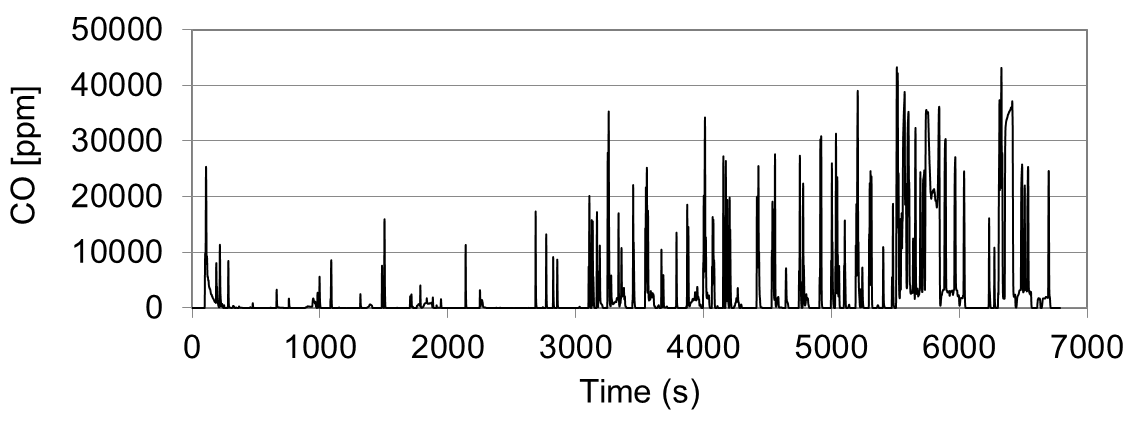


Tables S1. Emission factors of NOx (mg/km), NO_2_ (mg/km), CO (mg/km), CO_2_ (g/km) and PN (#/km) for the tested vehicles D1-D10, GV1-GV8 and CNG during the complete on-road tests (C.) and the sub-sections: urban (U.), rural (R.) and motorway (MW). T (°C) represents the ambient average temperature during the trip.

DV1

|  | **RDE-1** | | | |  | **RDE-2** | | | |  | **RDE-1-Dyn** | | | |  | **RDE-2-Dyn** | | | |  | **City-MW** | | | |  | **Hill** |
| --- | --- | --- | --- | --- | --- | --- | --- | --- | --- | --- | --- | --- | --- | --- | --- | --- | --- | --- | --- | --- | --- | --- | --- | --- | --- | --- |
| **T (°C)** | 17 | | | |  | 18 | | | |  | 15 | | | |  | 19 | | | |  |  | | | |  | 13 |
|  | **C.** | **U.** | **R.** | **MW** |  | **C.** | **U.** | **R.** | **MW** |  | **C.** | **U.** | **R.** | **MW.** |  | **C.** | **U.** | **R.** | **MW.** |  | **C.** | **U.** | **R.** | **MW.** |  | **C./Urb** |
| **NOx** | 474 | 279 | 385 | 869 |  | 477 | 296 | 336 | 872 |  | 646 | 525 | 494 | 973 |  | 759 | 523 | 433 | 1326 |  |  |  |  |  |  | 639 |
| **NO_2_** | 120 | 90 | 127 | 157 |  | 141 | 113 | 140 | 178 |  | 187 | 149 | 157 | 269 |  | 165 | 156 | 130 | 207 |  |  |  |  |  |  | 236 |
| **CO** | 262 | 258 | 151 | 409 |  | 405 | 349 | 235 | 666 |  | 298 | 378 | 266 | 228 |  | 188 | 197 | 104 | 256 |  |  |  |  |  |  | 205 |
| **CO_2_** | 187 | 220 | 143 | 196 |  | 189 | 218 | 135 | 210 |  | 204 | 257 | 150 | 197 |  | 202 | 229 | 145 | 223 |  |  |  |  |  |  | 381 |
| **PN ×10^11^** |  |  |  |  |  |  |  |  |  |  |  |  |  |  |  |  |  |  |  |  |  |  |  |  |  |  |

DV2-LCV

|  | **RDE-1** | | | |  | **RDE-2** | | | |  | **RDE-1-Dyn** | | | |  | **RDE-2-Dyn** | | | |  | **City-MW** | | | |  | **Hill** |
| --- | --- | --- | --- | --- | --- | --- | --- | --- | --- | --- | --- | --- | --- | --- | --- | --- | --- | --- | --- | --- | --- | --- | --- | --- | --- | --- |
| **T (°C)** | 26 | | | |  | 27 | | | |  | 27 | | | |  | 30 | | | |  | 30 | | | |  | 28 |
|  | **C.** | **U.** | **R.** | **MW** |  | **C.** | **U.** | **R.** | **MW** |  | **C.** | **U.** | **R.** | **MW.** |  | **C.** | **U.** | **R.** | **MW.** |  | **C.** | **U.** | **R.** | **MW.** |  | **C./Urb** |
| **NOx** | 483 | 260 | 525 | 775 |  | 620 | 472 | 515 | 884 |  | 628 | 392 | 679 | 886 |  | 939 | 682 | 872 | 1252 |  | 799 | 810 | 615 | 837 |  | 405 |
| **NO_2_** | 108 | 97 | 151 | 74 |  | 139 | 186 | 190 | 42 |  | 94 | 125 | 101 | 46 |  | 155 | 213 | 190 | 71 |  | 207 | 466 | 191 | 58 |  | 97 |
| **CO** | 30 | 63 | 10 | 2 |  | 20 | 40 | 4 | 7 |  | 38 | 48 | 37 | 28 |  | 12 | 22 | 2 | 10 |  | 32 | 81 | 19 | 5 |  | 129 |
| **CO_2_** | 134 | 151 | 106 | 141 |  | 144 | 152 | 108 | 163 |  | 153 | 169 | 136 | 153 |  | 155 | 167 | 131 | 160 |  | 145 | 157 | 118 | 145 |  | 155 |
| **PN ×10^11^** |  |  |  |  |  |  |  |  |  |  |  |  |  |  |  |  |  |  |  |  |  |  |  |  |  |  |

DV3

|  | **RDE-1** | | | |  | **RDE-2** | | | |  | **RDE-1-Dyn** | | | |  | **RDE-2-Dyn** | | | |  | **City-MW** | | | |  | **Hill** |
| --- | --- | --- | --- | --- | --- | --- | --- | --- | --- | --- | --- | --- | --- | --- | --- | --- | --- | --- | --- | --- | --- | --- | --- | --- | --- | --- |
| **T (°C)** | 15 | | | |  | 10 | | | |  | 10 | | | |  | 12 | | | |  | 11 | | | |  | 9 |
|  | **C.** | **U.** | **R.** | **MW** |  | **C.** | **U.** | **R.** | **MW** |  | **C.** | **U.** | **R.** | **MW.** |  | **C.** | **U.** | **R.** | **MW.** |  | **C.** | **U.** | **R.** | **MW.** |  | **C./Urb** |
| **NOx** | 551 | 412 | 433 | 882 |  | 586 | 445 | 414 | 893 |  | 998 | 907 | 770 | 1378 |  | 1011 | 762 | 904 | 1376 |  | 673 | 496 | 659 | 762 |  | 641 |
| **NO_2_** | 117 | 81 | 97 | 191 |  | 136 | 103 | 105 | 199 |  | 191 | 156 | 153 | 281 |  | 188 | 131 | 165 | 271 |  | 139 | 88 | 164 | 161 |  | 113 |
| **CO** | 28 | 39 | 23 | 20 |  | 21 | 33 | 8 | 17 |  | 106 | 155 | 48 | 106 |  | 44 | 83 | 46 | 1 |  | 34 | 78 | 8 | 17 |  | 103 |
| **CO_2_** | 154 | 156 | 135 | 173 |  | 156 | 153 | 128 | 181 |  | 164 | 173 | 145 | 174 |  | 165 | 156 | 151 | 186 |  | 158 | 147 | 120 | 169 |  | 148 |
| **PN ×10^11^** | 0.03 | 0.06 | <0.01 | <0.01 |  | <0.01 | <0.01 | <0.01 | <0.01 |  | 0.01 | <0.01 | <0.01 | <0.01 |  | <0.01 | <0.01 | <0.01 | <0.01 |  | <0.01 | <0.01 | <0.01 | <0.01 |  | 0.04 |

DV4

|  | **RDE-1** | | | |  | **RDE-2** | | | |  | **RDE-1-Dyn** | | | |  | **RDE-2-Dyn** | | | |  | **City-MW** | | | |  | **Hill** |
| --- | --- | --- | --- | --- | --- | --- | --- | --- | --- | --- | --- | --- | --- | --- | --- | --- | --- | --- | --- | --- | --- | --- | --- | --- | --- | --- |
| **T (°C)** | 24 | | | |  | 20 | | | |  | 18 | | | |  | 24 | | | |  | 26 | | | |  | 19 |
|  | **C.** | **U.** | **R.** | **MW** |  | **C.** | **U.** | **R.** | **MW** |  | **C.** | **U.** | **R.** | **MW.** |  | **C.** | **U.** | **R.** | **MW.** |  | **C.** | **U.** | **R.** | **MW.** |  | **C./U.** |
| **NOx** | 146 | 122 | 143 | 182 |  | 147 | 124 | 168 | 156 |  | 684 | 673 | 749 | 643 |  | 605 | 661 | 748 | 448 |  | 179 | 100 | 156 | 230 |  | 203 |
| **NO_2_** | 42 | 32 | 40 | 56 |  | 41 | 32 | 47 | 46 |  | 162 | 146 | 186 | 159 |  | 152 | 160 | 189 | 117 |  | 38 | 26 | 37 | 46 |  | 53 |
| **CO** | 5 | 4 | 8 | 4 |  | 5 | 5 | 6 | 3 |  | 9 | 11 | 7 | 10 |  | 3 | 0 | 0 | 9 |  | 9 | 5 | 1 | 13 |  | 4 |
| **CO_2_** | 160 | 193 | 132 | 146 |  | 155 | 179 | 131 | 147 |  | 174 | 212 | 147 | 155 |  | 172 | 209 | 139 | 161 |  | 152 | 170 | 111 | 152 |  | 173 |
| **PN ×10^11^** |  |  |  |  |  |  |  |  |  |  |  |  |  |  |  |  |  |  |  |  |  |  |  |  |  |  |

DV5

|  | **RDE-1** | | | |  | **RDE-2** | | | |  | **RDE-1-Dyn** | | | |  | **RDE-2-Dyn** | | | |  | **City-MW** | | | |  | **Hill** |
| --- | --- | --- | --- | --- | --- | --- | --- | --- | --- | --- | --- | --- | --- | --- | --- | --- | --- | --- | --- | --- | --- | --- | --- | --- | --- | --- |
| **T (°C)** | 13 | | | |  | 16 | | | |  | 14 | | | |  | 17 | | | |  |  | | | |  | 15 |
|  | **C.** | **U.** | **R.** | **MW** |  | **C.** | **U.** | **R.** | **MW** |  | **C.** | **U.** | **R.** | **MW.** |  | **C.** | **U.** | **R.** | **MW.** |  | **C.** | **U.** | **R.** | **MW.** |  | **C./U.** |
| **NOx** | 21 | 22 | 17 | 25 |  | 44 | 39 | 9 | 79 |  | 28 | 30 | 22 | 31 |  | 141 | 130 | 86 | 203 |  |  |  |  |  |  | 59 |
| **NO_2_** | 5 | 5 | 3 | 5 |  | 8 | 6 | 2 | 15 |  | 4 | 5 | 3 | 4 |  | 15 | 14 | 7 | 23 |  |  |  |  |  |  | 16 |
| **CO** | 45 | 26 | 93 | 22 |  | 25 | 29 | 35 | 12 |  | 20 | 33 | 4 | 21 |  | 51 | 45 | 115 | 0 |  |  |  |  |  |  | 57 |
| **CO_2_** | 157 | 188 | 131 | 140 |  | 155 | 189 | 128 | 140 |  | 170 | 235 | 122 | 139 |  | 183 | 247 | 145 | 154 |  |  |  |  |  |  | 194 |
| **PN ×10^11^** | 0.03 | 0.03 | 0.03 | 0.02 |  | 0.3 | 0.6 | 0.08 | 0.07 |  | 1.5 | 3.8 | 0.3 | 0.2 |  | - | - | - | - |  |  |  |  |  |  | - |

DV6

|  | **RDE-1** | | | |  | **RDE-2** | | | |  | **RDE-1-Dyn** | | | |  | **RDE-2-Dyn** | | | |  | **City-MW** | | | |  | **Hill** |
| --- | --- | --- | --- | --- | --- | --- | --- | --- | --- | --- | --- | --- | --- | --- | --- | --- | --- | --- | --- | --- | --- | --- | --- | --- | --- | --- |
| **T (°C)** | 11 | | | |  | 12 | | | |  | 25 | | | |  | 27 | | | |  | 23 | | | |  | 10 |
|  | **C.** | **U.** | **R.** | **MW** |  | **C.** | **U.** | **R.** | **MW** |  | **C.** | **U.** | **R.** | **MW.** |  | **C.** | **U.** | **R.** | **MW.** |  | **C.** | **U.** | **R.** | **MW.** |  | **C./Urb** |
| **NOx** | 75 | 89 | 45 | 88 |  | 81 | 84 | 81 | 77 |  | 376 | 325 | 367 | 449 |  | 224 | 256 | 162 | 248 |  | 141 | 131 | 88 | 173 |  | 93 |
| **NO_2_** | 21 | 23 | 18 | 22 |  | 23 | 28 | 22 | 19 |  | 126 | 107 | 109 | 168 |  | 82 | 78 | 56 | 111 |  | 67 | 47 | 40 | 92 |  | 38 |
| **CO** | 26 | 57 | 5 | 6 |  | 0 | 0 | 0 | 0 |  | 0 | 0 | 0 | 0 |  | 60 | 199 | 0 | 0 |  | 21 | 43 | 11 | 12 |  | 4 |
| **CO_2_** | 150 | 178 | 127 | 134 |  | 149 | 175 | 133 | 133 |  | 156 | 186 | 138 | 139 |  | 168 | 228 | 120 | 146 |  | 137 | 163 | 110 | 134 |  | 197 |
| **PN ×10^11^** | 5.4 | 8.5 | 4.4 | 1.9 |  | 4.6 | 7.6 | 4.0 | 1.9 |  | 6.1 | 8.4 | 5.4 | 4.0 |  | 6.0 | 13 | 2.5 | 1.6 |  | 3.4 | 6.2 | 3.13 | 1.8 |  | - |

DV7

|  | **RDE-1** | | | |  | **RDE-2** | | | |  | **RDE-1-Dyn** | | | |  | **RDE-2-Dyn** | | | |  | **City-MW** | | | |  | **Hill** |
| --- | --- | --- | --- | --- | --- | --- | --- | --- | --- | --- | --- | --- | --- | --- | --- | --- | --- | --- | --- | --- | --- | --- | --- | --- | --- | --- |
| **T (°C)** | 24 | | | |  | 28 | | | |  | 20 | | | |  | 27 | | | |  | 27 | | | |  | 23 |
|  | **C.** | **U.** | **R.** | **MW** |  | **C.** | **U.** | **R.** | **MW** |  | **C.** | **U.** | **R.** | **MW.** |  | **C.** | **U.** | **R.** | **MW.** |  | **C.** | **U.** | **R.** | **MW.** |  | **C./Urb** |
| **NOx** | 17 | 30 | 9 | 7 |  | 9 | 13 | 4 | 9 |  | 40 | 67 | 23 | 23 |  | 38 | 75 | 24 | 12 |  | 13 | 25 | 23 | 6 |  | 31 |
| **NO_2_** | 0 | 1 | 0 | 0 |  | 1 | 1 | 1 | 1 |  | 2 | 5 | 0 | 0 |  | 2 | 2 | 1 | 1 |  | 2 | 3 | 3 | 2 |  | 1 |
| **CO** | 40 | 52 | 29 | 36 |  | 0 | 0 | 0 | 0 |  | 49 | 65 | 36 | 41 |  | 4 | 10 | 0 | 0 |  | 30 | 45 | 27 | 23 |  | 65 |
| **CO_2_** | 151 | 182 | 120 | 142 |  | 153 | 178 | 121 | 151 |  | 152 | 182 | 122 | 145 |  | 153 | 185 | 122 | 148 |  | 135 | 147 | 116 | 133 |  | 162 |
| **PN ×10^11^** | 0.05 | 0.09 | 0.03 | 0.02 |  | 0.06 | 0.07 | 0.03 | 0.07 |  | 0.3 | 0.3 | 0.3 | 0.1 |  | 0.1 | 0.2 | 0.2 | 0.1 |  | 0.3 | 0.7 | 1 | 0.6 |  | 0.03 |

DV8

|  | **RDE-1** | | | |  | **RDE-2** | | | |  | **RDE-1-Dyn** | | | |  | **RDE-2-Dyn** | | | |  | **City-MW** | | | |  | **Hill** |
| --- | --- | --- | --- | --- | --- | --- | --- | --- | --- | --- | --- | --- | --- | --- | --- | --- | --- | --- | --- | --- | --- | --- | --- | --- | --- | --- |
| **T (°C)** | 27 | | | |  | 26 | | | |  | 30 | | | |  | 24 | | | |  | 28 | | | |  | 30 |
|  | **C.** | **U.** | **R.** | **MW** |  | **C.** | **U.** | **R.** | **MW** |  | **C.** | **U.** | **R.** | **MW.** |  | **C.** | **U.** | **R.** | **MW.** |  | **C.** | **U.** | **R.** | **MW.** |  | **C./Urb** |
| **NOx** | 31 | 35 | 26 | 29 |  | 89 | 61 | 71 | 138 |  | 318 | 252 | 267 | 464 |  | 57 | 101 | 29 | 31 |  | 19 | 29 | 13 | 15 |  | 72 |
| **NO_2_** | 4 | 4 | 2 | 6 |  | 8 | 3 | 7 | 15 |  | 23 | 15 | 17 | 40 |  | 4 | 3 | 4 | 4 |  | 0 | 0 | 0 | 0 |  | 0 |
| **CO** | 0 | 12 | 0 | 0 |  | 40 | 57 | 22 | 33 |  | 6 | 6 | 3 | 10 |  | 55 | 73 | 40 | 47 |  | 0 | 0 | 0 | 0 |  | 25 |
| **CO_2_** | 134 | 144 | 113 | 138 |  | 143 | 155 | 117 | 151 |  | 159 | 184 | 127 | 162 |  | 141 | 155 | 112 | 150 |  | 131 | 126 | 103 | 138 |  | 140 |
| **PN ×10^11^** | 0.7 | 0.7 | 0.7 | 0.5 |  | 0.6 | 0.6 | 0.5 | 0.5 |  | 0.7 | 1 | 0.6 | 0.5 |  | 0.7 | 0.8 | 0.6 | 0.5 |  | 0.6 | 0.7 | 0.6 | 0.6 |  | 0.5 |

DV9

|  | **RDE-1** | | | |  | **RDE-2** | | | |  | **RDE-1-Dyn** | | | |  | **RDE-2-Dyn** | | | |  | **City-MW** | | | |  | **Hill** |
| --- | --- | --- | --- | --- | --- | --- | --- | --- | --- | --- | --- | --- | --- | --- | --- | --- | --- | --- | --- | --- | --- | --- | --- | --- | --- | --- |
| **T (°C)** | 24 | | | |  | 29 | | | |  | 22 | | | |  | 22 | | | |  | 30 | | | |  | 27 |
|  | **C.** | **U.** | **R.** | **MW** |  | **C.** | **U.** | **R.** | **MW** |  | **C.** | **U.** | **R.** | **MW.** |  | **C.** | **U.** | **R.** | **MW.** |  | **C.** | **U.** | **R.** | **MW.** |  | **C./Urb** |
| **NOx** | 59 | 70 | 43 | 58 |  | 54 | 63 | 41 | 57 |  | 321 | 327 | 318 | 316 |  | 317 | 382 | 225 | 326 |  | 34 | 46 | 40 | 26 |  | 51 |
| **NO_2_** | 17 | 22 | 12 | 15 |  | 8 | 10 | 6 | 8 |  | 40 | 39 | 37 | 44 |  | 39 | 43 | 26 | 45 |  | 0 | 0 | 0 | 0 |  | 1 |
| **CO** | 8 | 8 | 1 | 15 |  | 0 | 0 | 0 | 0 |  | 0 | 0 | 0 | 0 |  | 0 | 0 | 0 | 0 |  | 0 | 0 | 0 | 0 |  | 0 |
| **CO_2_** | 188 | 221 | 156 | 178 |  | 188 | 215 | 155 | 187 |  | 210 | 247 | 171 | 203 |  | 208 | 259 | 160 | 193 |  | 171 | 196 | 150 | 160 |  | 202 |
| **PN ×10^11^** | 0.02 | 0.03 | 0.02 | 0.02 |  | 0.02 | 0.03 | 0.02 | 0.02 |  | 0.06 | 0.08 | 0.05 | 0.05 |  | 0.06 | 0.1 | 0.04 | 0.03 |  | <0.01 | <0.01 | <0.01 | <0.01 |  | 0.02 |

DV10

|  | **RDE-1** | | | |  | **RDE-2** | | | |  | **RDE-1-Dyn** | | | |  | **RDE-2-Dyn** | | | |  | **City-MW** | | | |  | **Hill** |
| --- | --- | --- | --- | --- | --- | --- | --- | --- | --- | --- | --- | --- | --- | --- | --- | --- | --- | --- | --- | --- | --- | --- | --- | --- | --- | --- |
| **T (°C)** | 24 | | | |  | 28 | | | |  | 27 | | | |  | 21 | | | |  | 27 | | | |  |  |
|  | **C.** | **U.** | **R.** | **MW** |  | **C.** | **U.** | **R.** | **MW** |  | **C.** | **U.** | **R.** | **MW.** |  | **C.** | **U.** | **R.** | **MW.** |  | **C.** | **U.** | **R.** | **MW.** |  | **C./Urb** |
| **NOx** | 92 | 32 | 39 | 242 |  | 145 | 47 | 47 | 337 |  | 385 | 316 | 236 | 627 |  | 290 | 273 | 219 | 376 |  | 89 | 26 | 67 | 136 |  |  |
| **NO_2_** | 22 | 7 | 11 | 55 |  | 31 | 8 | 12 | 71 |  | 125 | 94 | 88 | 202 |  | 94 | 74 | 93 | 119 |  | 16 | 0 | 15 | 30 |  |  |
| **CO** | 47 | 45 | 36 | 64 |  | 23 | 5 | 11 | 51 |  | 40 | 42 | 39 | 40 |  | 8 | 0 | 1 | 35 |  | 56 | 41 | 20 | 74 |  |  |
| **CO_2_** | 169 | 199 | 137 | 162 |  | 169 | 197 | 138 | 162 |  | 165 | 196 | 134 | 157 |  | 159 | 186 | 126 | 157 |  | 151 | 175 | 123 | 142 |  |  |
| **PN ×10^11^** | 0.03 | 0.03 | 0.02 | 0.02 |  | 0.02 | 0.02 | 0.01 | 0.02 |  | 0.1 | 0.2 | 0.06 | 0.04 |  | 0.04 | 0.06 | 0.04 | 0.03 |  | 0.01 | 0.02 | 0.01 | 0.01 |  |  |

GV1

|  | **RDE-1** | | | |  | **RDE-2** | | | |  | **RDE-1-Dyn** | | | |  | **RDE-2-Dyn** | | | |  | **City-MW** | | | |  | **Hill** |
| --- | --- | --- | --- | --- | --- | --- | --- | --- | --- | --- | --- | --- | --- | --- | --- | --- | --- | --- | --- | --- | --- | --- | --- | --- | --- | --- |
| **T (°C)** | 26 | | | |  | 26 | | | |  | 28 | | | |  | 21 | | | |  | 27 | | | |  |  |
|  | **C.** | **U.** | **R.** | **MW** |  | **C.** | **U.** | **R.** | **MW** |  | **C.** | **U.** | **R.** | **MW.** |  | **C.** | **U.** | **R.** | **MW.** |  | **C.** | **U.** | **R.** | **MW.** |  | **C./Urb** |
| **NOx** | 6 | 8 | 7 | 3 |  | 6 | 9 | 5 | 2 |  | 14 | 25 | 10 | 2 |  | 11 | 18 | 10 | 5 |  | 9 | 11 | 13 | 5 |  | 8 |
| **NO_2_** | 0 | 0 | 0 | 0 |  | 0 | 0 | 0 | 0 |  | 0 | 0 | 0 | 0 |  | 0 | 0 | 0 | 0 |  | 0 | 0 | 0 | 0 |  | 0 |
| **CO** | 699 | 352 | 766 | 1127 |  | 662 | 447 | 172 | 1360 |  | 1494 | 645 | 1927 | 2249 |  | 4163 | 2484 | 4581 | 5709 |  | 370 | 315 | 264 | 439 |  | 1046 |
| **CO_2_** | 158 | 151 | 149 | 179 |  | 152 | 152 | 127 | 174 |  | 171 | 179 | 155 | 177 |  | 162 | 151 | 153 | 183 |  | 163 | 165 | 120 | 175 |  | 140 |
| **PN ×10^11^** | 3.9 | 3.1 | 4.2 | 4.8 |  | 7.4 | 6.5 | 6.1 | 9.5 |  | 11 | 10 | 11 | 12 |  | 11 | 12 | 10 | 10 |  | 2.6 | 1.8 | 2.6 | 3.0 |  | 10 |

GV2

|  | **RDE-1** | | | |  | **RDE-2** | | | |  | **RDE-1-Dyn** | | | |  | **RDE-2-Dyn** | | | |  | **City-MW** | | | |  | **Hill** |
| --- | --- | --- | --- | --- | --- | --- | --- | --- | --- | --- | --- | --- | --- | --- | --- | --- | --- | --- | --- | --- | --- | --- | --- | --- | --- | --- |
| **T (°C)** | 12 | | | |  | 7 | | | |  | 5 | | | |  | 9 | | | |  | 8 | | | |  | 5 |
|  | **C.** | **U.** | **R.** | **MW** |  | **C.** | **U.** | **R.** | **MW** |  | **C.** | **U.** | **R.** | **MW.** |  | **C.** | **U.** | **R.** | **MW.** |  | **C.** | **U.** | **R.** | **MW.** |  | **C./Urb** |
| **NOx** | 13 | 20 | 6 | 10 |  | 16 | 19 | 8 | 20 |  | 13 | 19 | 10 | 6 |  | 11 | 16 | 7 | 9 |  | 11 | 15 | 9 | 9 |  | 19 |
| **NO_2_** | 2 | 2 | 1 | 2 |  | 4 | 4 | 3 | 4 |  | 3 | 3 | 3 | 4 |  | 3 | 2 | 2 | 3 |  | 3 | 3 | 3 | 3 |  | 3 |
| **CO** | 3344 | 149 | 1011 | 10910 |  | 1039 | 242 | 343 | 2666 |  | 6727 | 3078 | 4598 | 14101 |  | 5741 | 1979 | 3930 | 11443 |  | 2234 | 145 | 773 | 4121 |  | 450 |
| **CO_2_** | 154 | 188 | 119 | 146 |  | 157 | 186 | 122 | 150 |  | 155 | 196 | 122 | 135 |  | 149 | 169 | 123 | 148 |  | 135 | 156 | 108 | 129 |  | 166 |
| **PN ×10^11^** | 2.0 | 3.9 | 0.6 | 0.8 |  |  |  |  |  |  | 5.4 | 10 | 2.6 | 1.8 |  | 3.5 | 6.7 | 1. | 1.1 |  | 1.7 | 3.9 | 0.7 | 0.5 |  | 5.1 |

GV3

|  | **RDE-1** | | | |  | **RDE-2** | | | |  | **RDE-1-Dyn** | | | |  | **RDE-2-Dyn** | | | |  | **City-MW** | | | |  | **Hill** |
| --- | --- | --- | --- | --- | --- | --- | --- | --- | --- | --- | --- | --- | --- | --- | --- | --- | --- | --- | --- | --- | --- | --- | --- | --- | --- | --- |
| **T (°C)** | 12 | | | |  | 22 | | | |  | 19 | | | |  | 30 | | | |  | 15 | | | |  | 18 |
|  | **C.** | **U.** | **R.** | **MW** |  | **C.** | **U.** | **R.** | **MW** |  | **C.** | **U.** | **R.** | **MW.** |  | **C.** | **U.** | **R.** | **MW.** |  | **C.** | **U.** | **R.** | **MW.** |  | **C./Urb** |
| **NOx** | 38 | 73 | 19 | 7 |  | 39 | 58 | 50 | 7 |  | 25 | 41 | 23 | 6 |  | 41 | 91 | 20 | 6 |  | 21 | 53 | 7 | 5 |  | 56 |
| **NO_2_** | 0 | 1 | 0 | 0 |  | 1 | 1 | 1 | 1 |  | 0 | 0 | 0 | 0 |  | 0 | 1 | 0 | 0 |  | 0 | 0 | 0 | 0 |  | 3 |
| **CO** | 170 | 149 | 202 | 162 |  | 245 | 223 | 156 | 350 |  | 1398 | 382 | 1831 | 2255 |  | 1057 | 368 | 1782 | 1146 |  | 455 | 185 | 724 | 561 |  | 167 |
| **CO_2_** | 135 | 166 | 105 | 126 |  | 123 | 111 | 120 | 139 |  | 152 | 183 | 127 | 135 |  | 145 | 156 | 131 | 145 |  | 137 | 153 | 99 | 134 |  | 156 |
| **PN ×10^11^** | 11 | 20 | 51 | 7.2 |  | 12 | 12 | 9.4 | 15 |  | 25 | 46 | 9.9 | 14 |  | 31 | 65 | 11 | 13 |  | 8.9 | 11 | 5.0 | 8.7 |  | 19 |

GV4

|  | **RDE-1** | | | |  | **RDE-2** | | | |  | **RDE-1-Dyn** | | | |  | **RDE-2-Dyn** | | | |  | **City-MW** | | | |  | **Hill** |
| --- | --- | --- | --- | --- | --- | --- | --- | --- | --- | --- | --- | --- | --- | --- | --- | --- | --- | --- | --- | --- | --- | --- | --- | --- | --- | --- |
| **T (°C)** | 24 | | | |  | 20 | | | |  | 17 | | | |  | 24 | | | |  | 26 | | | |  | 19 |
|  | **C.** | **U.** | **R.** | **MW** |  | **C.** | **U.** | **R.** | **MW** |  | **C.** | **U.** | **R.** | **MW.** |  | **C.** | **U.** | **R.** | **MW.** |  | **C.** | **U.** | **R.** | **MW.** |  | **C./Urb** |
| **NOx** | 98 | 119 | 152 | 11 |  | 84 | 142 | 91 | 11 |  | 91 | 189 | 36 | 18 |  | 95 | 223 | 31 | 11 |  | 62 | 124 | 112 | 8 |  | 288 |
| **NO_2_** | 2 | 2 | 2 | 2 |  | 2 | 2 | 1 | 2 |  | 3 | 4 | 2 | 3 |  | 2 | 3 | 2 | 3 |  | 2 | 3 | 2 | 2 |  | 2 |
| **CO** | 1022 | 364 | 667 | 2323 |  | 958 | 405 | 354 | 2115 |  | 1838 | 1613 | 1446 | 2594 |  | 3315 | 3232 | 2266 | 4350 |  | 930 | 264 | 1391 | 1238 |  | 199 |
| **CO_2_** | 133 | 149 | 117 | 126 |  | 147 | 165 | 121 | 149 |  | 165 | 214 | 129 | 140 |  | 162 | 200 | 131 | 146 |  | 132 | 149 | 113 | 125 |  | 167 |
| **PN ×10^11^** | 22 | 28 | 15 | 22 |  | 25 | 28 | 16 | 30 |  | 37 | 56 | 25 | 27 |  | 31 | 43 | 21 | 28 |  | 21 | 22 | 14 | 22 |  | 27 |

GV5

|  | **RDE-1** | | | |  | **RDE-2** | | | |  | **RDE-1-Dyn** | | | |  | **RDE-2-Dyn** | | | |  | **City-MW** | | | |  | **Hill** |
| --- | --- | --- | --- | --- | --- | --- | --- | --- | --- | --- | --- | --- | --- | --- | --- | --- | --- | --- | --- | --- | --- | --- | --- | --- | --- | --- |
| **T (°C)** | 26 | | | |  | 31 | | | |  | 30 | | | |  | 30 | | | |  | 24 | | | |  | 25 |
|  | **C.** | **U.** | **R.** | **MW** |  | **C.** | **U.** | **R.** | **MW** |  | **C.*** | **U.** | **R.** | **MW.** |  | **C.** | **U.** | **R.** | **MW.** |  | **C.** | **U.** | **R.** | **MW.** |  | **C./Urb** |
| **NOx** | 13 | 17 | 10 | 12 |  | 9 | 18 | 2 | 4 |  | 23 | 44 | 15 | 5 |  | 17 | 33 | 13 | 3 |  | 5 | 10 | 3 | 3 |  | 10 |
| **NO_2_** | 0 | 0 | 0 | 0 |  | 1 | 2 | 1 | 1 |  | 2 | 2 | 1 | 2 |  | 1 | 2 | 1 | 1 |  | 1 | 1 | 0 | 1 |  | 1 |
| **CO** | 257 | 137 | 146 | 561 |  | 376 | 77 | 113 | 970 |  | 1078 | 364 | 752 | 2425 |  | 650 | 439 | 301 | 1200 |  | 108 | 55 | 56 | 145 |  | 115 |
| **CO_2_** | 151 | 184 | 116 | 143 |  | 157 | 191 | 118 | 149 |  | 172 | 217 | 136 | 155 |  | 164 | 208 | 127 | 154 |  | 140 | 162 | 114 | 134 |  | 142 |
| **PN ×10^11^** | 1 | 0.1 | 0.8 | 0.8 |  | 0.8 | 1.2 | 0.6 | 0.6 |  | 1.3 | 1.7 | 1.2 | 1.1 |  | 1.1 | 1.4 | 1.0 | 1.0 |  | 1.0 | 1.3 | 1.0 | 0.8 |  | 1.0 |

GV6

|  | **RDE-1** | | | |  | **RDE-2** | | | |  | **RDE-1-Dyn** | | | |  | **RDE-2-Dyn** | | | |  | **City-MW** | | | |  | **Hill** |
| --- | --- | --- | --- | --- | --- | --- | --- | --- | --- | --- | --- | --- | --- | --- | --- | --- | --- | --- | --- | --- | --- | --- | --- | --- | --- | --- |
| **T (°C)** | 25 | | | |  | 30 | | | |  | 18 | | | |  | 19 | | | |  |  | | | |  |  |
|  | **C.** | **U.** | **R.** | **MW** |  | **C.** | **U.** | **R.** | **MW** |  | **C.** | **U.** | **R.** | **MW.** |  | **C.** | **U.** | **R.** | **MW.** |  | **C.** | **U.** | **R.** | **MW.** |  | **C./Urb** |
| **NOx** | 20 | 21 | 5 | 35 |  | 22 | 48 | 7 | 5 |  | 16 | 33 | 5 | 4 |  | 16 | 38 | 2 | 5 |  |  |  |  |  |  |  |
| **NO_2_** | 0 | 0 | 0 | 0 |  | 0 | 0 | 0 | 0 |  | 0 | 0 | 0 | 0 |  | 0 | 0 | 0 | 0 |  |  |  |  |  |  |  |
| **CO** | 1050 | 973 | 524 | 1739 |  | 925 | 455 | 569 | 1764 |  | 8601 | 8245 | 5237 | 12837 |  | 6501 | 6309 | 4580 | 8472 |  |  |  |  |  |  |  |
| **CO_2_** | 214 | 273 | 173 | 174 |  | 188 | 217 | 146 | 192 |  | 222 | 281 | 173 | 197 |  | 208 | 256 | 161 | 199 |  |  |  |  |  |  |  |
| **PN ×10^11^** | 18 | 18 | 11 | 25 |  | 24 | 32 | 16 | 21 |  | 110 | 130 | 79 | 110 |  | 97 | 120 | 67 | 98 |  |  |  |  |  |  |  |

GV7

|  | **RDE-1** | | | |  | **RDE-2** | | | |  | **RDE-1-Dyn** | | | |  | **RDE-2-Dyn** | | | |  | **City-MW** | | | |  | **Hill** |
| --- | --- | --- | --- | --- | --- | --- | --- | --- | --- | --- | --- | --- | --- | --- | --- | --- | --- | --- | --- | --- | --- | --- | --- | --- | --- | --- |
| **T (°C)** | 27 | | | |  | 32 | | | |  | 28 | | | |  | 33 | | | |  | 29 | | | |  | 30 |
|  | **C.** | **U.** | **R.** | **MW** |  | **C.** | **U.** | **R.** | **MW** |  | **C.** | **U.** | **R.** | **MW.** |  | **C.** | **U.** | **R.** | **MW.** |  | **C.** | **U.** | **R.** | **MW.** |  | **C./Urb** |
| **NOx** | 60 | 101 | 37 | 27 |  | 31 | 52 | 13 | 18 |  | 113 | 195 | 72 | 51 |  | 70 | 143 | 37 | 21 |  | 18 | 31 | 50 | 7 |  | 25 |
| **NO_2_** | 1 | 1 | 1 | 0 |  | 0 | 0 | 0 | 0 |  | 4 | 4 | 3 | 5 |  | 0 | 0 | 0 | 0 |  | 0 | 0 | 0 | 0 |  | 0 |
| **CO** | 252 | 80 | 102 | 660 |  | 614 | 76 | 21 | 1751 |  | 3971 | 2125 | 4840 | 5436 |  | 2635 | 2961 | 3610 | 1500 |  | 307 | 102 | 346 | 412 |  | 127 |
| **CO_2_** | 164 | 206 | 132 | 138 |  | 167 | 211 | 122 | 147 |  | 179 | 236 | 133 | 155 |  | 181 | 253 | 133 | 147 |  | 154 | 191 | 131 | 137 |  | 186 |
| **PN ×10^11^** | 27 | 33 | 17 | 32 |  | 34 | 31 | 12 | 54 |  | 67 | 81 | 54 | 64 |  | 57 | 89 | 39 | 39 |  | 18 | 20 | 15 | 17 |  | 19 |

GV8

|  | **RDE-1** | | | |  | **RDE-2** | | | |  | **RDE-1-Dyn** | | | |  | **RDE-2-Dyn** | | | |  | **City-MW** | | | |  | **Hill** |
| --- | --- | --- | --- | --- | --- | --- | --- | --- | --- | --- | --- | --- | --- | --- | --- | --- | --- | --- | --- | --- | --- | --- | --- | --- | --- | --- |
| **T (°C)** | 22 | | | |  | 23 | | | |  | 29 | | | |  | 23 | | | |  | 26 | | | |  | 23 |
|  | **C.** | **U.** | **R.** | **MW** |  | **C.** | **U.** | **R.** | **MW** |  | **C.*** | **U.** | **R.** | **MW.** |  | **C.** | **U.** | **R.** | **MW.** |  | **C.** | **U.** | **R.** | **MW.** |  | **C./Urb** |
| **NOx** | 24 | 34 | 11 | 24 |  | 15 | 25 | 3 | 14 |  | 205 | 251 | 314 | 25 |  | 25 | 32 | 14 | 26 |  | 16 | 25 | 11 | 12 |  | 18 |
| **NO_2_** | 2 | 2 | 1 | 1 |  | 1 | 2 | 1 | 1 |  | 12 | 16 | 16 | 3 |  | 2 | 2 | 1 | 2 |  | 2 | 2 | 1 | 2 |  | 3 |
| **CO** | 189 | 41 | 83 | 520 |  | 133 | 26 | 17 | 353 |  | 2408 | 1118 | 2861 | 3625 |  | 3065 | 766 | 2422 | 6102 |  | 181 | 23 | 17 | 294 |  | 149 |
| **CO_2_** | 173 | 210 | 135 | 162 |  | 170 | 188 | 135 | 178 |  | 179 | 214 | 145 | 170 |  | 189 | 223 | 156 | 183 |  | 158 | 167 | 132 | 160 |  | 182 |
| **PN ×10^11^** | 0.2 | 0.2 | 0.1 | 0.2 |  | 0.2 | 0.2 | 0.1 | 0.2 |  | 0.2 | 0.3 | 0.2 | 0.2 |  | 0.4 | 0.4 | 0.3 | 0.5 |  | 0.1 | 0.1 | 0.1 | 0.2 |  | 0.1 |

*During a second repetition of the same dynamic test the emissions factors for the complete test were: NOx 23mg/km, NO_2_ 2mg/km, CO 3973mg/km, CO_2_ 190g/km, PN 5×10^10^ #/km

CNG-LCV

|  | **RDE-1** | | | |  | **RDE-2** | | | |  | **RDE-1-Dyn** | | | |  | **RDE-2-Dyn** | | | |  | **City-MW** | | | |  | **Hill** |
| --- | --- | --- | --- | --- | --- | --- | --- | --- | --- | --- | --- | --- | --- | --- | --- | --- | --- | --- | --- | --- | --- | --- | --- | --- | --- | --- |
| **T (°C)** | 26 | | | |  | 25 | | | |  | 25 | | | |  | 29 | | | |  | 29 | | | |  | 31 |
|  | **C.** | **U.** | **R.** | **MW** |  | **C.** | **U.** | **R.** | **MW** |  | **C.** | **U.** | **R.** | **MW.** |  | **C.** | **U.** | **R.** | **MW.** |  | **C.** | **U.** | **R.** | **MW.** |  | **C./Urb** |
| **NOx** | 308 | 641 | 123 | 42 |  | 354 | 702 | 253 | 47 |  | 852 | 1718 | 527 | 89 |  | 1060 | 1673 | 1052 | 307 |  | 242 | 659 | 178 | 51 |  | 515 |
| **NO_2_** | 15 | 23 | 9 | 9 |  | 15 | 26 | 11 | 7 |  | 27 | 55 | 13 | 6 |  | 39 | 63 | 34 | 12 |  | 16 | 32 | 11 | 9 |  | 21 |
| **CO** | 369 | 270 | 274 | 612 |  | 245 | 111 | 155 | 478 |  | 187 | 123 | 119 | 349 |  | 230 | 195 | 203 | 298 |  | 440 | 150 | 203 | 635 |  | 317 |
| **CO_2_** | 256 | 296 | 211 | 249 |  | 231 | 277 | 181 | 223 |  | 287 | 342 | 240 | 269 |  | 277 | 323 | 227 | 267 |  | 251 | 277 | 206 | 248 |  | 251 |
| **PN ×10^11^** | 11 | 26 | 0.6 | 0.2 |  | 1.6 | 3.7 | 0.4 | 0.3 |  |  |  |  |  |  |  |  |  |  |  |  |  |  |  |  |  |
